# Cooperation in group-structured populations with two layers of interactions

**DOI:** 10.1038/srep17446

**Published:** 2015-12-03

**Authors:** Yanling Zhang, Feng Fu, Xiaojie Chen, Guangming Xie, Long Wang

**Affiliations:** 1 School of Automation and Electrical Engineering, University of Science and Technology Beijing, Beijing 100083, China; 2 Theoretical Biology, Institute of Integrative Biology, ETH Zürich, 8092 Zürich, Switzerland; 3 School of Mathematical Sciences, University of Electronic Science and Technology of China, Chengdu 611731, China; 4 Center for Systems and Control, State Key Laboratory for Turbulence and Complex Systems, College of Engineering, Peking University, Beijing 100871, China

## Abstract

Recently there has been a growing interest in studying multiplex networks where individuals are structured in multiple network layers. Previous agent-based simulations of games on multiplex networks reveal rich dynamics arising from interdependency of interactions along each network layer, yet there is little known about analytical conditions for cooperation to evolve thereof. Here we aim to tackle this issue by calculating the evolutionary dynamics of cooperation in group-structured populations with two layers of interactions. In our model, an individual is engaged in two layers of group interactions simultaneously and uses unrelated strategies across layers. Evolutionary competition of individuals is determined by the total payoffs accrued from two layers of interactions. We also consider migration which allows individuals to move to a new group within each layer. An approach combining the coalescence theory with the theory of random walks is established to overcome the analytical difficulty upon local migration. We obtain the exact results for all “isotropic” migration patterns, particularly for migration tuned with varying ranges. When the two layers use one game, the optimal migration ranges are proved identical across layers and become smaller as the migration probability grows.

The evolution of cooperation has gained increasing attention from evolutionary biologists and physicists. Evolutionary game theory provides a powerful mathematical setting for this problem and has led to many deep insights thereof. An important way of resolving the dilemma of cooperation is network reciprocity^1^,^2^,^3^,^4^,^5^, which has been put into the spotlight by games in graph-structured^6^,^7^,^8^,^9^,^10^,^11^,^12^,^13^,^14^,^15^,^16^,^17^,^18^,^19^ and in group-structured populations^20^,^21^,^22^,^23^,^24^,^25^,^26^. In graph-structured populations, each node represents an individual and games happen between connected individuals. Yet in group-structured populations, each group holds a sub-population which is divided by geographical sites or phenotypic tags, and games generally occur between individuals of the same group. Most explorations of games in structured populations have performed on single-layered networks^9^,^10^,^11^,^12^,^13^,^22^,^23^,^24^,^25^,^26^,^27^,^28^, where individuals are structured in a single network layer. Recently some studies have shifted attention towards multiplex networks^16^,^17^,^18^,^19^, where individuals are structured in multiple network layers. In these investigations of games on multiplex networks, agent-based simulations reveal rich dynamics arising from interdependency of interactions along each network layer, yet there is little known about the exact analytical condition for cooperation to evolve thereof.

Multiplex networks explain that an individual has different kinds of ties defined by social properties, such as working for a particular company, living in a specific location, or going to a certain university. We propose a minimal model of multiplex networks by considering group-structured populations with two layers of interactions. In our model (Fig. 1A), each individual is located in two network layers simultaneously and uses unrelated strategies (cooperation or defection) across layers. In each layer, *M* groups (geographical islands or phenotypic tags) are arranged in a regular circle and individuals can play games if and only if they are in one group (there occur no games in the one-player group). Across layers, individuals can play one game (the prisoner’s dilemma or the snowdrift game) or different games (the prisoner’s dilemma vs. the snowdrift game). In the prisoner’s dilemma, the benefit *b* > 0 is gained from the cooperative opponent, and the cost *c* > 0 is paid by the cooperator. In the snowdrift game, the benefit *b* > 0 is gained if at least one side cooperates, and the cost *c* > 0 is divided equally between cooperators. In this paper, the critical value of *c*/*b* for natural selection to favor cooperation over defection is our focus.

In each update, one individual is chosen randomly and equiprobably to die and one competes to reproduce an offspring with probability proportional to the fitness, which is determined by the sum of the payoffs obtained in the two layers just like the previous study^17^. Here we do not consider the general case where the fitness is determined by a weighted sum of the payoffs in the two layers^18^,^19^, but checked that the evolutionary outcomes are unchanged substantially for the general case. The newborn offspring adopts the strategies of the parent in the two layers with probability 1 − *u*, otherwise mutates to one strategy randomly and equiprobably within each layer. Meanwhile, the newborn offspring stays in the groups of the parent in the two layers with probability 1 − *v*, otherwise migrates to a new group within each layer according to the prespecified migration pattern.

To better understand the prespecified migration pattern, we show it by a two-dimensional lattice consisting of the integer points in [1, *M*]^2^ and satisfying the periodic boundary condition (*i*_1_ + *l*_1_*M*, *i*_2_ + *l*_2_*M*) = (*i*_1_, *i*_2_) where *l*_1_ and *l*_2_ are integers. The abscissa and the ordinate denote the group space of the first layer and the one of the second, respectively. An edge exists between two points if and only if there is a potential single-step migration path between them. In other words, the offspring can migrate to one of the points connected to the point where the parent resides. The lattices which look the same from every point are our focus, whose corresponding migration patterns have been called “isotropic” in the population genetics^29^. For simplicity, “isotropic” migration patterns of one network layer are illustrated in Fig. 1B. In this paper, we obtain the exact analytic condition for cooperation to evolve under all “isotropic” migration patterns, and analyze the optimal migration ranges of the two layers leading to the largest critical cost-to-benefit ratio. When the two layers use the same game, the optimal migration ranges are proved identical across layers and become smaller as the migration probability grows. When the two layers use different games, they can be different across layers.

A series of recent theoretical findings paves a way for quantifying the evolutionary dynamics in group-structured populations^22^,^23^,^24^,^25^,^26^,^27^,^28^. These studies investigate the largest and the smallest migration range in the single-layered group structure, which mean that individuals can disperse from any one to any other group and between only groups that are nearest neighbors, respectively. In these studies, the coalescence theory is used alone to deal with the largest migration range, and combined with the theory of a particular random walk to tackle the smallest^22^,^24^. However, none of migration patterns have been analyzed theoretically except the above two types in single-layered, let alone in multiplex networks. In this paper, we provide a method combining the coalescence theory with the theory of general random walks to overcome the analytical difficulty upon local migration in two-layered and in single-layered networks. The key point of our method is that we find the probability that the walker of all random walks moves from one to any point after *t* steps following the example of the previous study^30^, and thus the method holds for all migration patterns from local to global migration. Moreover in our method, the theory of random walks is employed to trace both the migration and the mutation process, and thus the method not only solves local migration, but also prepares for analyzing local mutation theoretically.

## Results

The cooperative level of the whole system *x*_*C*_ is defined as


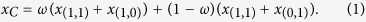



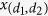
 is the frequency of the strategy (*d*_1_, *d*_2_) where *d*_1_, *d*_2_ ∈ {0, 1} (1 for cooperation and 0 for defection) are the strategy of the first layer and the one of the second, respectively. The parameter *ω* ∈ [0, 1] specifies the proportion of the cooperative level of the first layer in the overall cooperative level. For *ω* = 0.5, *x*_*C*_ is measured in the traditional way^17^,^18^,^19^, in which the cooperative behavior of each layer plays an equal role in measuring *x*_*C*_. *ω* = 1 or *ω* = 0 means that the cooperative level of only the first or of only the second determines *x*_*C*_.

Natural selection favors cooperation over defection if the cooperative level of the whole system predominates in the stationary distribution, i.e., 〈*x*_*C*_〉 > 1/2 where 〈*x*_*C*_〉 is the mean *x*_*C*_ in the stationary distribution. Under weak selection, which means the fitness differences between individuals are small, the condition for cooperation to evolve can be calculated analytically. Weak selection is often used in population genetics^31^,^32^ and suggested in the adaptive dynamics^33^,^34^, as the mutant strategy is drawn from an infinitesimally small neighborhood around the resident strategy so that the fitness differences are very small.

### General migration patterns

Cooperation is favored by natural selection if the cost-to-benefit ratio *c*/*b* is below the critical one (*c*/*b*)^*^ which is obtained when 〈*x*_*C*_〉 = 0.5. Larger (*c*/*b*)^*^ means that cooperation is more favored. The expression of (*c*/*b*)^*^ for *ω* ∈ [0, 1] is


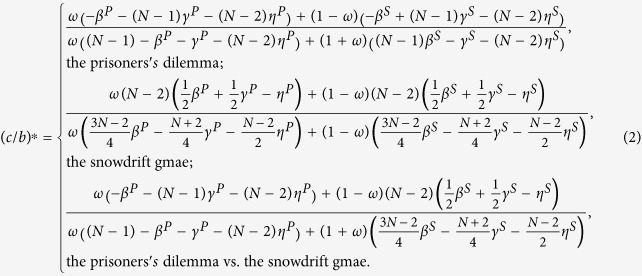


The above *β*^*P*^, *γ*^*P*^, *η*^*P*^, *β*^*S*^, *γ*^*S*^, and *η*^*S*^ describe the strategy-and-location distribution of multiple individuals in the neutral selection: when two individuals are chosen without replacement, *β*^*P*^ (*β*^*S*^) is the chance that they reside in one group of the first layer (of the second), and *γ*^*P*^ (*γ*^*S*^) is the probability that they have both the same strategies in the two layers and the same group of the first layer (of the second); when three individuals are chosen without replacement, *η*^*P*^ (*η*^*S*^) is the probability that the former two have the same strategies in the two layers and the latter two are in one group of the first layer (of the second).

A method combining the coalescence theory with the theory of random walks in spatial lattices is established to calculate *β*^*P*^, *γ*^*P*^, *η*^*P*^, *β*^*S*^, *γ*^*S*^, and *η*^*S*^. The method can be divided into two steps: from the present (the time when multiple individuals are chosen) backwards to the time of their most recent common ancestor (MRCA), the coalescence theory is used to acquire the distribution about the number of migration and of mutation events in the ancestral process; from the time of MRCA forwards to the present, the random walk is employed to trace the changing path of the strategy and of the location upon each lineage. Considering all possible states weighted by the corresponding probabilities, we finally accomplish the calculation of the strategy-and-location distribution of multiple individuals and further obtain


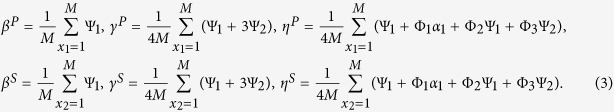


Note that for simplicity, the functions Ψ_1_, Ψ_2_, Φ_1_, Φ_2_, and Φ_3_ omit (*f*(*x*_1_, *M*)) under 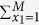 (in *β*^*P*^, *γ*^*P*^, and *η*^*P*^) and (*f*(*M*, *x*_2_)) under 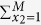 (in *β*^*S*^, *γ*^*S*^, and *η*^*S*^), 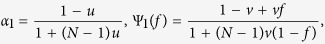 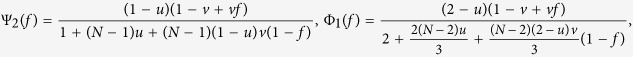 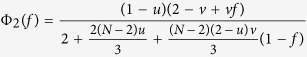, and 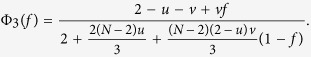 The expression of *f*(*x*_1_, *x*_2_) is 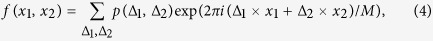


where *p*(Δ_1_, Δ_2_) is the probability that a single-step migration results in the displacement vector (Δ_1_, Δ_2_) and *i* is the imaginary unit satisfying *i*^2^ = −1. The function *f*(*x*_1_, *x*_2_), which corresponds to the structured function of random walks on the two-dimensional lattice^30^, carries the full information of the migration pattern. It appears in *β*^*P*^, *γ*^*P*^, and *η*^*P*^ with the form of the single variable function *f*(*x*_1_, *M*) and in *β*^*S*^, *γ*^*S*^, and *η*^*S*^ with *f*(*M*, *x*_2_). Obviously, *f*(*x*_1_, *M*) and *f*(*M*, *x*_2_) characterize the displacement distribution in the first and in the second layer that a single-step migration leads to, respectively. Eventually, substituting Eq. (3) into (2), we obtain the concrete value of (*c*/*b*)^*^ (shown in Methods), which applies to all migration patterns from local to global migration by adjusting the function *f*(*x*_1_, *x*_2_). Besides, there are no approximations in calculating (*c*/*b*)^*^, and thus its value holds for any population size, any mutation probability, any migration probability, and any group number in each layer.

In our model, an individual takes part in two layers of interactions simultaneously and the strategy in a given layer updates according to the total payoffs obtained in the two layers. Such model is called the two-layered network model accordingly. As the benchmark, the single-layered network model is investigated, in which an individual participates in interactions of only the first layer or of only the second and the update of the strategy in the single layer is up to the payoff obtained from only that layer. Obviously, the cooperative level of the single-layered network model coincides with Eq. (1) when *ω* = 1 (when *ω* = 0).

For *ω* = 1 (for *ω* = 0), the condition for cooperation in the two-layered network model coincides with the one in the single-layered. Although the cooperative levels in these two models are exactly the same when *ω* = 1 (when *ω* = 0), this coincidence is not seemingly obvious, as the update of the strategy in a given layer is different in them. This coincidence is because the payoff obtained in one layer imposes a vanishing effect on the evolution of the strategy in the other when *ω* = 1 (when *ω* = 0). The critical cost-to-benefit ratio (*c*/*b*)^*^ of the single-layered network model is the one for *ω* = 1 (for *ω* = 0) in Eq. (2) when individuals participate in interactions of only the first layer (of only the second). It is proved to be a decreasing function of the mutation probability *u*, and thus cooperation is more favored for the smaller mutation probability.

### Migration tuned by varying ranges

**Figure 2:**
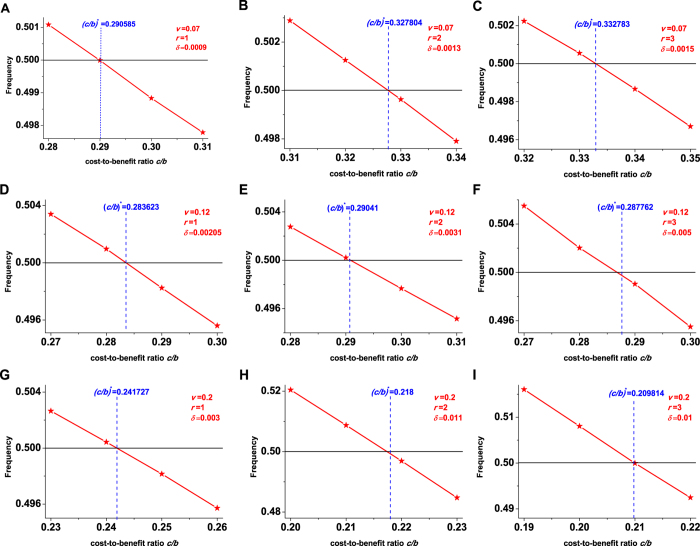
Agreement between analytic calculations and simulation results. A population of size *N* = 50 is distributed in the single-layered network consisting of *M* = 7 groups. Individuals play the prisoner’s dilemma with others in the same group. Each point indicates the frequency of cooperators averaged over around 5 × 10^9^ generations. Decreasing the cost-to-benefit ratio *c*/*b* favors cooperators. The critical cost-to-benefit ratio (*c*/*b*)^*^ (intersection with the horizontal line) is obtained when 〈*x*_*C*_〉 = 1/2. We study three migration ranges *r* = 1, *r* = 2, and *r* = 3 for three migration probabilities *v* = 0.07, *v* = 0.12, and *v* = 0.2. The optimal migration range r^*^ leads to the largest value of (*c*/*b*)^*^ over the set 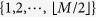. When *v* = 0.07, r^*^ = 3. When *v* = 0.12, r^*^ = 2. When *v* = 0.2, r^*^ = 1. The mutation probability *u* = 0.01, the benefit *b* = 1, and the selection intensity *δ* and the cost *c* vary accordingly.

In each network layer, *M* groups are arranged in a circle, and thus the distance between two groups takes on one of the values 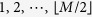 where 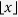 is the largest integer less than or equal to *x*. Here, we focus on a representative type of migration patterns determined by the migration ranges of the two layers *r*_1_ and *r*_2_, which represent the largest displacement in the first and in the second layer that a single-step migration leads to, respectively. In Fig. 2B, we illustrate the migration pattern of each network layer characterized by the migration range *r*. All possible displacement vectors that a single-step migration leads to are assumed to form the set Ω(*r*_1_) × Ω(*r*_2_) where Ω(*r*) = {1, 2, ···, *r*}, which suggests that an individual disperses in the two layers simultaneously. In addition, all elements of Ω(*r*_1_) × Ω(*r*_2_) are assumed to be performed equiprobably. The corresponding *f*(*x*_1_, *x*_2_) is equal to *f*(*x*_1_; *r*_1_)*f*(*x*_2_; *r*_2_) where 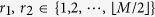, 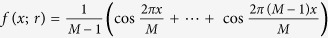 if 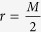 for even *M* and 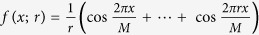 otherwise. It is noteworthy that the reason why the terms sin(···) disappear in *f*(*x*_1_, *x*_2_) is the symmetry of the migration to the left and to the right direction. Substituting *f*(*x*_1_, *M*) = *f*(*x*_1_; *r*_1_) and *f*(*M*, *x*_2_) = *f*(*x*_2_; *r*_2_) into Eq. (3) and (2), we obtain (*c*/*b*)^*^ when the migration ranges of the two layers are *r*_1_ and *r*_2_.

In the following, we mean the case 0 < *ω* < 1 by the two-layered network model without particular emphasis. In the two-layered network model, the critical cost-to-benefit ratio (*c*/*b*)^*^ depends on migration ranges and game types of the two layers. Given the migration probability *v* and the mutation probability *u*, for two cases in which the two network layers use the same and different games, we will show the optimal migration ranges 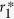 and 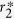 which lead to the largest value of (*c*/*b*)^*^ over the set Ω(*r*_1_) × Ω(*r*_2_).

When the two network layers use one game, it has been proved that the optimal migration ranges of the two layers 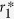 and 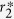 are both equal to the one of the single-layered network model, which holds for any *ω* ∈ (0, 1). Accordingly, the division of the plane (*v*, *u*) regarding 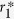 and 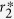 is in line with that of the single-layered network model and identical for all *ω* ∈ (0, 1). The results of the single-layered network model are in agreement with numerical simulations (Fig. 2).

**Figure 3:**
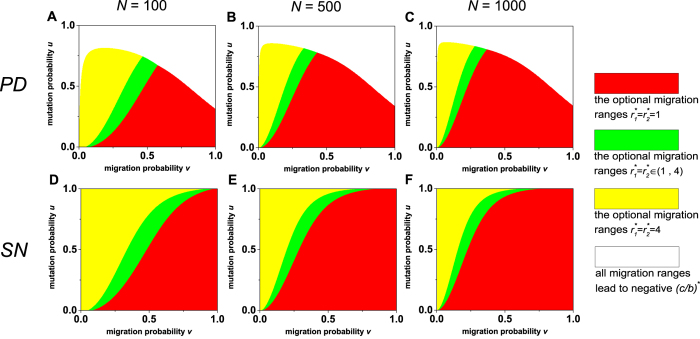
When the two network layers use the same game, the division of the plane (*v*, *u*) based on the optimal migration ranges of the two layers 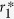 and 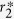, which lead to the largest value of the critical cost-to-benefit ratio (*c*/*b*)^*^ over the set {1, 2, ⋯, ⌊*M*/2⌋} × {1, 2, ⋯, ⌊*M*/2⌋}. A population of size *N* = 100, *N* = 500, or *N* = 1000 is distributed in two network layers, each of which assumes *M* = 9 groups. The proportion of the cooperative level of the first layer in the overall cooperative level *ω* = 0.5. The two layers use the prisoner’s dilemma (PD) in (**A–C**) and the snowdrift game (SN) in (**D–F**).

In Fig. 3A–C, the case in which the two network layers use the prisoner’s dilemma is investigated. For small migration probabilities *v* or large mutation probabilities *u*, any migration ranges of the two layers can’t induce natural selection to favor cooperation over defection. For other probabilities *u* and *v*, the optimal migration ranges of the two layers 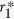 and 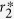 are both identical to the value of *r* which can be the largest (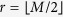), some intermediate 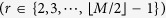, or the smallest range (*r* = 1). As *v* grows, 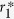 and 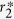 become smaller. In Fig. 3D–F, the case in which the two layers use the snowdrift game is studied. The phenomenon disappears that any migration ranges of the two layers can’t lead natural selection to favor cooperation. The optimal migration ranges 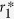 and 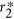 are still identical and are located successively in the largest, some intermediate, and the smallest range as *v* grows, just like the case for the prisoner’s dilemma. The division of the plane (*v*, *u*) based on 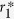 and 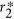 is slightly affected by the population size *N* and greatly affected by the group number of each layer *M* (Fig. S1 in Supplementary Information).

The following is the explanation as to why the optimal migration ranges of the two layers become smaller as the migration probability grows. The small migration probability prevents cooperators from finding empty groups. In a group full of cooperators and defectors, cooperators will be overcome by defectors and possible to survive if they leave the current group and find empty groups. Here, the largest migration range provides most opportunities for a migratory cooperator to find an empty group and is best in promoting cooperation. Whereas the large migration probability synchronizes the population (makes individuals move in a more well-mixed environment), raising the likelihood all cooperators are overcome by defectors. In this case, the smallest migration range imposes the largest limitation on the exploitation of cooperators (defectors can exploit cooperators in only their own and nearest neighbor groups) and maximizes the cooperator frequency. When the migration probability is moderate, some intermediate migration range, which brings the migratory cooperators enough opportunities of finding empty groups and restricts the exploitation of cooperators by defectors, is the most ideal.

**Figure 4:**
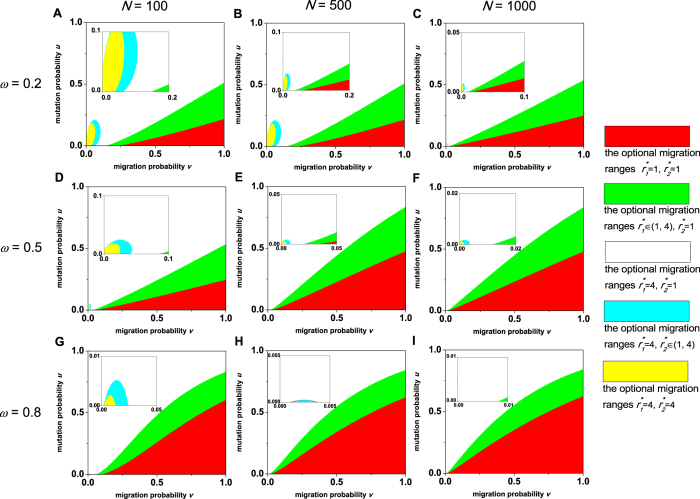
When the first layer uses the prisoner’s dilemma and the second the snowdrift game, the division of the plane (*v*, *u*) based on the optimal migration ranges of the two layers 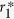 and 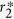, which lead to the largest value of the critical cost-to-benefit ratio (*c*/*b*)^*^ over the set {1, 2, ⋯, ⌊*M*/2⌋} × {1, 2, ⋯, ⌊*M*/2⌋}. A population of size *N* = 100, *N* = 500, or *N* = 1000 is distributed in two network layers, each of which assumes *M* = 9 groups. We study three cases *ω* = 0.2, *ω* = 0.5, and *ω* = 0.8, where *ω* is the proportion of the cooperative level of the first layer in the overall cooperative level. The insets show the division of the plain (*v*, *u*) more clearly when *v* and *u* are both small.

When the first layer uses the prisoner’s dilemma and the second the snowdrift game, the optimal migration ranges of the two layers 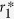 and 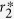 are not necessarily identical and the division of the plane (*v*, *u*) regarding 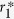 and 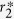 relies on *ω* (Fig. 4). As the migration probability *v* grows, 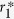 becomes smaller, and the appearance sequence of 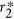 is related with the mutation probability *u*. This indicates the prisoner’s dilemma plays a predominant role in the evolution relative to the snowdrift game. As the population size *N* or *ω* increases, or as the group number of each layer *M* decreases (Fig. S2 in Supplementary Information), the area for 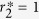 expands and even occupies the whole plane (*v*, *u*). This shows that the impact of the prisoner’s dilemma on the evolution becomes stronger compared to the one of the snowdrift game.

## Discussion

We provide a minimal model of multiplex networks, where an individual takes part in group interactions of two network layers simultaneously. The exact analytic condition for cooperation is calculated for two cases in which the two layers use one game (the prisoner’s dilemma or the snowdrift game) and different games (the prisoner’s dilemma vs. the snowdrift game), and holds for all “isotropic” migration patterns from local to global migration. In particular, a type of migration tuned with varying ranges is investigated to obtain the migration ranges of the two layers which are best in promoting cooperation.

Our study is similar to the previous^17^, as both assume that an individual takes part in interactions of more than one network layer simultaneously. There are, however, three obvious differences between these two studies. Our model assumes the more realistic group rather than graph structure in the previous^17^. Unlike the previous study^17^, where the cooperative behaviors of all network layers play the equal role in measuring the overall cooperative level, we introduce a parameter *ω* ∈ [0, 1] so that the cooperative behaviors of the two layers can be unequal. The parameter *ω* acts as a bridge between the two-layered and the single-layered network model in the sense that the conditions for cooperation in them are the same when *ω* = 1 or when *ω* = 0. Migration, as the central feature of ecosystems in reality, is drawing increasing interest in solving the dilemma of cooperation^9^,^10^,^11^,^12^,^13^,^20^,^21^,^22^. It is completely ignored in the previous study^17^, but introduced into the evolutionary dynamics in our study.

We provide an approach combining the coalescence theory with the theory of random walks in spatial lattices to overcome the analytic difficulty upon local migration in the two-layered group structure. As a byproduct, local migration in the single-layered group structure is analyzed theoretically by our method. In the previous theoretical work^22^,^24^, only migration of the smallest range, as an extreme case of local migration, has been examined in the single-layered group structure. Our method is similar to the previous^22^,^24^ in that both of them use the coalescence theory and the theory of random walks, whereas there are four significant differences between them. The previous method^22^,^24^ is mainly based on a particular random walk which has an explicit expression of the probability that the walker moves from one to any point after *t* steps, and thus holds for only migration of the smallest range. In contrast, the highlight of ours is that we find the unified expression of such probability for any random walk, and thus ours can deal with all migration patterns. Unlike the previous method^22^,^24^, which adopts the continuous-time approximation of the discrete coalescent process and the one of the discrete random walk, we make no approximations in using the coalescence theory and the theory of random walks, and thus our method holds for any population size, any mutation probability, any migration probability, and any group number of each layer. The analyses in the previous method^22^,^24^ are the same for the Moran and the Wright-Fisher process by assuming large population limit. Yet in our method, the analyses for the Moran process are more complicate than those for the Wright-Fisher by assuming any population size, and ours is applied to the Moran process in this paper. In contrast to the previous method^22^,^24^, in which the theory of random walks is used to follow only the migration process, we employ the theory of random walks to trace both the migration and the mutation process, and thus our method not only deals with local migration, but also paves a way for investigating local mutation theoretically.

The exact analytic condition for cooperation is illustrated by a type of migration tuned by varying ranges. When the two network layers use one game, the optimal migration ranges of the two layers have been proved to be both identical to the one of the single-layered network model and become smaller as the migration probability grows. When the two layers use different games, they can be different. In the single-layered network model, our analyses consider all possible migration ranges together and are more comprehensive than the previous^22^, which compare only the largest and the smallest migration range. Besides, we find a new result that some intermediate migration range is best in promoting cooperation for some moderate migration probability. Moreover, our method can also be used to study the evolution of cooperation based on tag^24^, where the group is viewed as the tag that helps cooperators to make the strategy, and can further extend such study for all patterns of tag mutation.

## Methods

### The condition for cooperation

In a finite population of size *N*, individual *i* endowed with the strategy vector 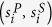 adopts 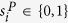 in the first layer and 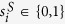 in the second (1 for cooperation and 0 for defection). Assuming that an individual interacts with any others in the same group of each layer, the payoff of individual *i* obtained in the first layer is denoted by 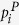 and the one in the second 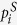. The fitness of individual *i* is 
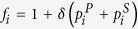
, where *δ* is the selection intensity.

The mean frequency of the strategy (1, 1) in the stationary distribution is calculated by the Mutation-Selection analysis as follows. In a single-step update, the frequency of the strategy (1, 1) denoted by *x*_(1,1)_ is increased in two ways: an existing individual with the strategy (1, 1) reproduces, and the offspring does not mutate to other strategies; an existing individual with other strategies reproduces, and the offspring mutates to the strategy (1, 1). Yet in a single-step update, there is only one way to decrease *x*_(1,1)_: an existing individual with the strategy (1, 1) dies. Thus, the expected change of *x*_(1,1)_ in a single-step update is 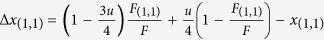 where *F*_(1,1)_ and *F* are the total fitness of individuals with the strategy (1, 1) and of the population, respectively. Since the mean Δ*x*_(1,1)_ in the stationary distribution is zero, then 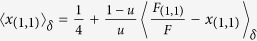 where 〈*X*〉 denotes the mean *X* in the stationary distribution and the subscript of 〈〉 is the selection intensity. Performing the perturbation theory in the limit of *δ* → 0, we have


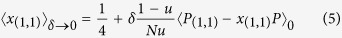


where *P*_(1,1)_ and *P* are the total payoffs of individuals with the strategy (1, 1) and of the population, respectively. Similarly under weak selection, the mean frequency of (1, 0) and of (0, 1) in the stationary distribution is 〈*x*_(1,0)_〉_*δ*→0_ and 〈*x*_(0,1)_〉_*δ*→0_ respectively:


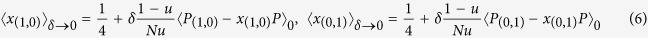


where *P*_(1,0)_ and *P*_(0,1)_ are the total payoffs of individuals with (1, 0) and with (0, 1), respectively. Eventually, the evolutionary dynamics under weak selection can be calculated on the basis of the neutral selection *δ* = 0, which assumes no fitness differences between individuals.

According to Eq. (1), (5), and (6), the mean *x*_*C*_ in the stationary distribution is


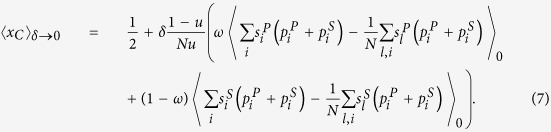


Therefore, the condition for natural selection to favor cooperation over defection is


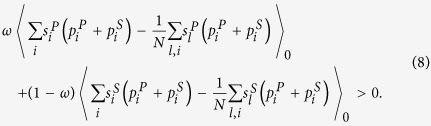


This condition in Eq. (8) reduces to (for detailed reduction process please refer to Supplementary Information)


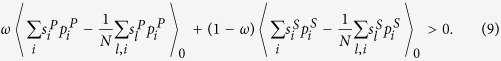


The detailed transformation of 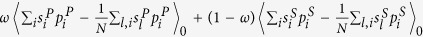 is shown in Supplementary Information. By letting it be 0, i.e., 〈*x*_*C*_〉 = 0.5, the corresponding *c*/*b* is denoted by (*c*/*b*)^*^:


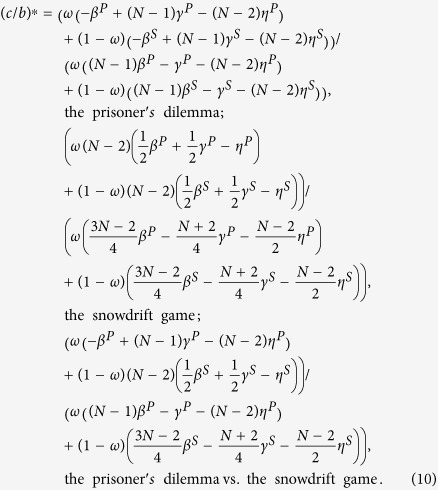


The above *β*^*P*^, *γ*^*P*^, *η*^*P*^, *β*^*S*^, *γ*^*S*^, and *η*^*S*^ describe the strategy-and-location distribution of multiple individuals in the neutral selection.

### A method combining the coalescence theory with the theory of random walks

In each update, there is a single newborn offspring who was reproduced and a single parent who reproduced in the immediately previous generation. Imagine that *k* individuals labelled by *I*_1_, *I*_2_, ···, *I*_*k*_ are chosen randomly and without replacement from the population to form a sample: with probability 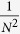, *I*_*x*_ and *I*_*y*_ of the sample are the newborn offspring and the parent, respectively (for detailed analysis please refer to Supplementary Information); with probability 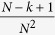, *I*_*x*_ of the sample is the newborn offspring and the rest are not the parent; with probability 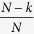, none of the sample is the newborn offspring. Obviously with probability 1/*N*^2^, the ancestor of *I*_*x*_ and the one of *I*_*y*_ are the newborn offspring and the parent respectively when the first coalescence of the sample happens. Given the first coalescence happens upon the lineages of *I*_*x*_ and *I*_*y*_ at the moment *T* by looking backwards from the present generation 0. During the former *T* − 1 moments between two generations 0 and *T* − 2, the probability of 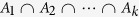 where *A*_*i*_ means that there appear *k*_*i*_ newborn offspring on the lineage of *I*_*i*_ is


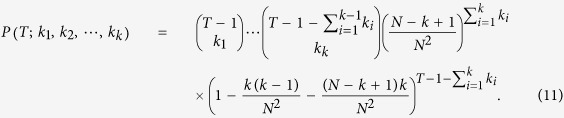


Based on Eq. (11), the probability of 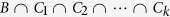 where *B* represents that the first coalescence of the sample happens upon the lineage of *I*_*x*_ and of *I*_*y*_ at the time *T* and *C*_*i*_ denotes that until the time *T* the lineage of *I*_*i*_ has *k*_*i*_ newborn offspring is


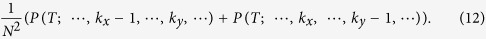


Considering the independence of migration and mutation upon a newborn offspring and the independence of migration or the independence of mutation in different generations, the probability of 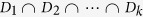 where *D*_*i*_ denotes that *g*_*i*_ migrate and *h*_*i*_ mutate among *k*_*i*_ newborn offspring is


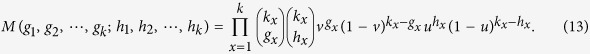


It is noteworthy that there are no approximations in the above coalescence theory and our results hold for any population size, any migration probability, and any mutation probability.

In our model, the location space is characterized by a two-dimensional lattice satisfying the periodic boundary condition (*a*_1_ + *j*_1_*M*, *a*_2_ + *j*_2_*M*) = (*a*_1_, *a*_2_) where *j*_1_ and *j*_2_ are integers. Given that the ancestor of *I*_*x*_ at the time *T* is located in *η*_*x*_ and until then *g*_*x*_ migration events occur upon the lineage of *I*_*x*_, the migration process along the lineage of *I*_*x*_ becomes a discrete random walk on the two-dimensional lattice. One-step migration paths and the corresponding probabilities describe the displacement distribution that a single step of the random walker leads to. Consequently, the probability that *I*_*x*_ is located in *γ*_*x*_ is


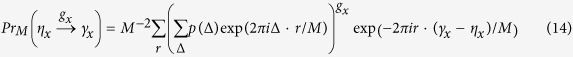


where *p*(Δ) is the probability that a single-step migration results in the location displacement Δ and *i* is the imaginary unit satisfying *i*^2^ = −1. The function 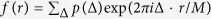, which corresponds to the structured function on the two-dimensional lattice with period *M*^30^, carries the full information of the migration pattern. Eq. (14) describes essentially the probability that a random walker moves from one to any point after *t* steps, and holds for all migration patterns. This equality is not given directly in the previous study, but can be derived following the example of the previous^30^. There are no approximations in the above calculations, and thus our result holds for any group number of each layer. Similarly, the strategy space is described as a one-dimensional lattice satisfying *b*_1_ + *j*_1_*S* = *b*_1_ where *j*_1_ is an integer. Given that the ancestor of *I*_*x*_ at the time *T* uses the strategy *θ*_*x*_ and until then *h*_*x*_ mutation events occur upon the lineage of *I*_*x*_, the mutation process along the lineage of *I*_*x*_ can be traced by a discrete random walk on such one-dimensional lattice. Eventually, the probability that *I*_*x*_ uses the strategy *δ*_*x*_ is


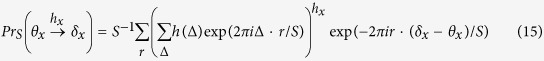


where *h*(Δ) is the probability that a single-step mutation results in the strategy change Δ. The function 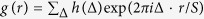 describes the full information of the mutation pattern. Note that our method paves the way for studying local mutation by adjusting *g*(*r*).

At the time of the first coalescence, the sample of *k* individuals have *k* − 1 ancestors and two of them have the same ancestor. The probability that the *k* − 1 individuals (ancestors) are located in *η*_1_, *η*_2_, ···, *η*_*k*−1_ respectively and use strategies *θ*_1_, *θ*_2_, ···, *θ*_*k*−1_ respectively is denoted by


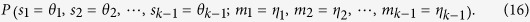


In our model, *η*_1_, *η*_2_, ···, *η*_*k*−1_ are points on the two-dimensional lattice with period *M* and *θ*_1_, *θ*_2_, ···, *θ*_*k*−1_ are points on the one-dimensional lattice with period 4. There are *k*(*k* − 1)/2 possible pairs of lineages for the sample to first coalesce into a common ancestor, and we assume that the first coalescence of the sample happens upon the lineage of *I*_*x*_ and the one of *I*_*y*_ in the following. *T*, which is the time that it takes the sample to reach their first coalescence, takes on one of the values 1, 2, ···. Meanwhile, *k*_1_, *k*_2_, ···, *k*_*k*_, where *k*_*i*_ is the number of newborn offspring along the ancestral lineage of *I*_*i*_, range from 0 to *T* and satisfy *k*_1_ + *k*_2_ + ··· *k*_*k*_ ≤ *T*. Moreover, *g*_1_ (*g*_2_, ···, *g*_*k*_) and *h*_1_ (*h*_2_, ···, *h*_*k*_), where *g*_*i*_ and *h*_*i*_ are the number of migration and of mutation events among *k*_*i*_ newborn offspring respectively, take on one value between 0 and *k*_1_ (*k*_2_, ···, *k*_*k*_). For simplicity, the common ancestor of *I*_*x*_ and *I*_*y*_ at the time *T* is assumed to be located in *η*_1_ and to use the strategy *θ*_1_, and the ancestors of the other *k* − 2 individuals of the sample at the time *T* are assumed to be located in *η*_2_, *η*_3_, ···, *η*_*k*−1_ respectively and to use strategies *θ*_2_, *θ*_3_, ···, *θ*_*k*−1_ respectively. Given the above conditions, according to Eq. (14) and (15), the probability that the *k* individuals are located in *γ*_1_, *γ*_2_, ···, *γ*_*k*_ respectively and use strategies *δ*_1_,*δ*_2_, ···,*δ*_*k*_ respectively is


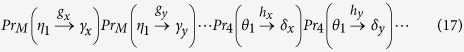


By considering all possible strategies and locations of *k* − 1 ancestors at the first coalescence in Eq. (16), all possible first coalescence pairs in Eq. (17), all possible values of *T* and of *k*_1_, *k*_2_, ···, *k*_*k*_ in Eq. (12), all possible values of *g*_1_, *g*_2_, ···, *g*_*k*_ and of *h*_1_, *h*_2_, ···, *h*_*k*_ in Eq. (13), and weighting the system in those states by the stationary probabilities, we obtain a recurrence relation between the strategy-and-location distribution of *k* individuals and the one of *k* − 1 individuals, whose initial condition is the known distribution of one individual’s strategy and location.

### The calculation of *β* ^*P*^, *γ* ^*P*^, *η* ^*P*^, *β* ^*S*^, *γ* ^*S*^, and *η* ^*S*^

Since the strategy-and-location distribution of an individual is known, we can obtain the values of *β*^*P*^, *γ*^*P*^, *β*^*S*^, and *γ*^*S*^ by considering the case *k* = 2 in the above analyses (for detailed calculation please refer to Supplementary Information):


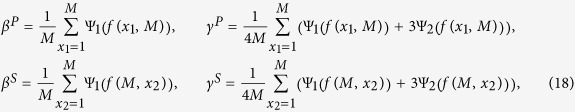


where 
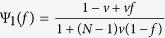
 and 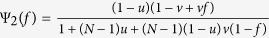. Meanwhile, we can obtain the values of *η*^*P*^ and *η*^*S*^ by considering the cases *k* = 3 and *k* = 2 in the above analyses (for detailed calculation please refer to Supplementary Information):


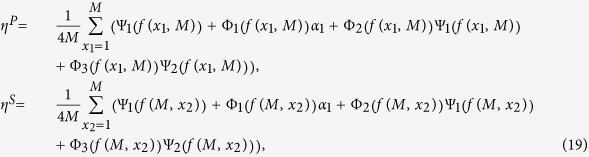


where 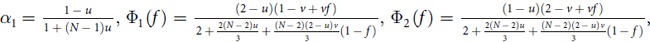 and 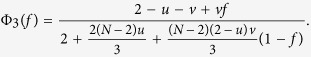 It is noteworthy that our method prepares for investigating three-person games by considering the cases *k* = 4, *k* = 3, and *k* = 2 in the above analyses.

Substituting Eq. (18) and (19) into Eq. (10), we have


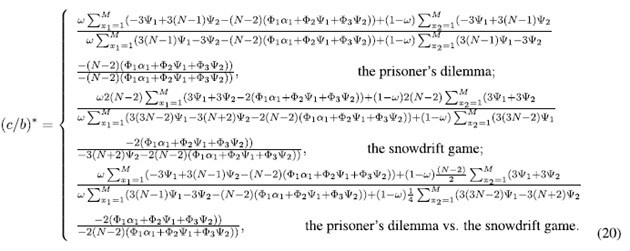



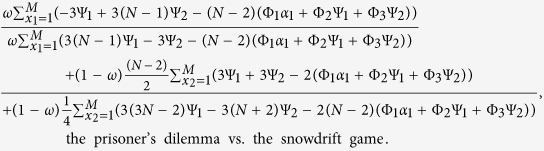


Note that the functions Ψ_1_, Ψ_2_, Φ_1_, Φ_2_, and Φ_3_ omit (*f*(*x*_1_, *M*)) under 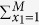 and (*f*(*M*, *x*_2_)) under 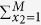 for simplicity.

### Single-layered network model

Following the example of the Mutation-Selection analysis and the perturbation theory used in the two-layered network model, the condition for cooperation in the single-layered network model is


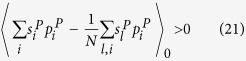


when individuals participate in interactions of only the first layer or is


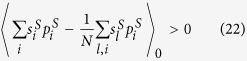


when only the second. Eq. (21) or (22) happens to be the condition for cooperation in the two-layered network model when *ω* = 1 or when *ω* = 0 (see Eq. (9)). Without loss of generality, we assume individuals participate in interactions of only the first layer. By letting *ω* in Eq. (20) be 1, we obtain the (*c*/*b*)^*^ of the single-layered network model,


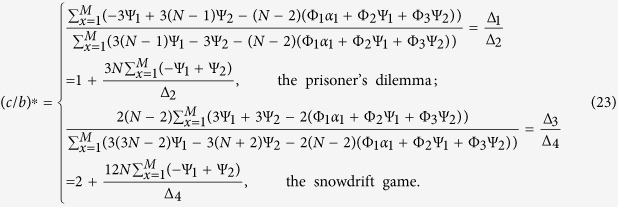


Note that the functions Ψ_1_, Ψ_2_, Φ_1_, Φ_2_, and Φ_3_ omit (*f*(*x*, *M*)). Since Δ_2_ and Δ_4_ are increasing functions with respect to *u* and equal to zero when *u* = 0, they are positive for all *u* ∈ (0, 1]. Then two values of (*c*/*b*)^*^ in Eq. (23) are decreasing functions with respect to *u* since −Ψ_1_ + Ψ_2_ decreases as *u* grows.

### Migration tuned by varying ranges

When the two network layers use the prisoner’s dilemma, the optimal migration ranges of the two layers are both equal to the one of the single-layered network model. The proof is as follows. Based on Eq. (20), (*c*/*b*)^*^ for *ω* = 1 (for *ω* = 0) varies with the migration range of only the first layer (of only the second) and is independent of the one of the other layer. When the migration ranges of the two layers are *r*_1_ and *r*_2_, (*c*/*b*)^*^ for *ω* = 1 (for *ω* = 0) is denoted by *P*(*r*_1_) (by *P*(*r*_2_)) and (*c*/*b*)^*^ for 0 < *ω* < 1 is *P*(*r*_1_, *r*_2_). According to Eq. (20), when *r*_1_ = *r*_2_, or when *r*_1_ ≠ *r*_2_ and *P*(*r*_1_) = *P*(*r*_2_),


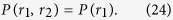


As (*c*/*b*)^*^ for 0 < *ω* < 1 is between (*c*/*b*)^*^ for *ω* = 1 and the one for *ω* = 0, when *r*_1_ = *r*_2_ and *P*(*r*_1_) = *P*(*r*_2_),


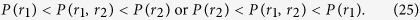


Let *P*(r^*^) be the maximum of *P*(*r*) over the set 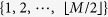, i.e., 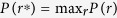, and let 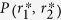 be the maximum of *P*(*r*_1_, *r*_2_) over the set 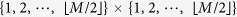, i.e., 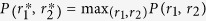. Eq. (24) shows when *r*_1_ = *r*_2_ = r^*^, *P*(*r*_1_, *r*_2_) reaches *P*(r^*^). Eq. (24) and (25) show when *r*_1_ ≠ *r*_2_, *P*(*r*_1_, *r*_2_) can not exceed *P*(r^*^). In summary, 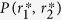 is equal to *P*(r^*^) and the optimal migration ranges of the two layers 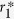 and 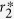 can be the same and equal to r^*^. Since the (*c*/*b*)^*^ for *ω* = 1 (for *ω* = 0) is equal to the (*c*/*b*)^*^ of the single-layered network model, the optimal migration ranges of the two layers are both equal to the one of the single-layered network model. When the two layers use the snowdrift game, the same conclusion can be obtained similarly. Note that the above proof does not specify the value of *ω* and holds for any *ω* ∈ (0, 1).

## Additional Information

**How to cite this article**: Zhang, Y. *et al.* Cooperation in group-structured populations with two layers of interactions. *Sci. Rep.* **5**, 17446; doi: 10.1038/srep17446 (2015).

## Supplementary Material

Supplementary Information

## Figures and Tables

**Figure 1:**
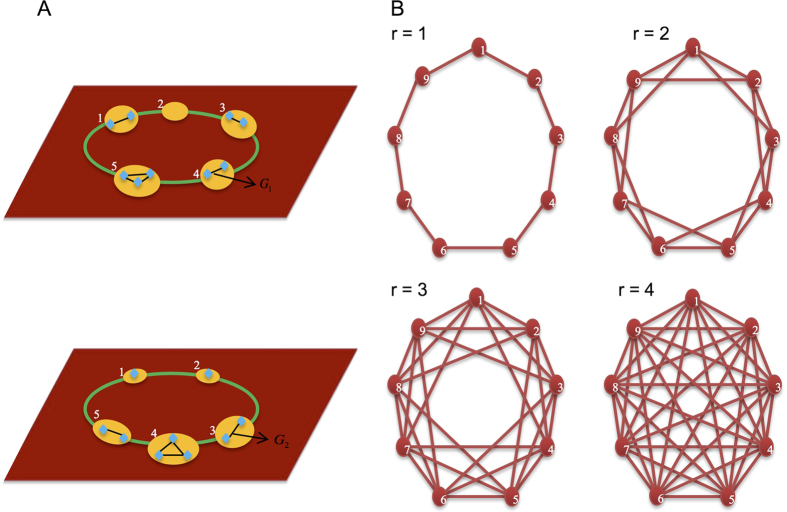
Evolution in group-structured populations with two layers of interactions. (**A**) Each individual (blue diamond) participates in interactions of two network layers simultaneously. In each layer, five groups (yellow oval) are arranged in a regular circle and labelled from 1 to 5 in clockwise. Each group allows any number of individuals to reside in (including no individuals). Individuals perform two games *G*_1_ and *G*_2_ with others who reside in the same group of the first and of the second layer, respectively. Reproduction of an individual depends on the total payoffs obtained in the two layers, and the offspring can migrate to a group within each layer according to the prespecified migration pattern. (**B**) “Isotropic” migration patterns in each network layer. Nine groups (red node) are arranged in a regular circle and labelled from 1 to 9 in clockwise. The edge exists between two nodes if and only if there is a potential single-step migration path between them. In other words, an offspring can migrate to one of the nodes connected to the node in which the parent is located. The distance between two groups takes on one of the values 1, 2, 3, 4. The migration range *r* means that the set of the displacements that a single-step migration leads to is Ω(*r*) = {1, …, *r*}.
