# Supplementary material for: Cooperation in group-structured populations with two layers of interactions
Source: Sci Rep. 2015 Dec 3;5:17446. doi: 10.1038/srep17446 (PMC4668372; doi:10.1038/srep17446)
Supplement: Supplementary Information [file srep17446-s1.pdf]

Supplementary Information:

Cooperation in group-structured populations with two layers of interactions

Yanling Zhang[1,4], Feng Fu[2], Xiaojie Chen[3], Guangming Xie[4*] & Long Wang[4]

[1] School of Automation and Electrical Engineering, University of Science and Technology Beijing, Beijing 100083, China

[2] Theoretical Biology, Institute of Integrative Biology, ETH Zürich, 8092 Zürich, Switzerland

[3] School of Mathematical Sciences, University of Electronic Science and Technology of China, Chengdu 611731, China

[4] Center for Systems and Control, State Key Laboratory for Turbulence and Complex Systems, College of Engineering, Peking University, Beijing 100871, China

Contents

I. The condition for cooperation 2

II. A method combining the coalescence theory with the theory of random walks 7

III. The calculation of β^P, γ^P, β^S and γ^S 7

IV. The calculation η^S and η^S 9

V. Figure S1 and S2 12

I. THE CONDITION FOR COOPERATION

In the main text, the condition for cooperation is given as

$$\omega\langle \sum_i s_i^P(p_i^P + p_i^S) - \frac{1}{N}\sum_{l,i} s_l^P(p_i^P + p_i^S)\rangle_0 + (1-\omega)\langle \sum_i s_i^S(p_i^P + p_i^S) - \frac{1}{N}\sum_{l,i} s_l^S(p_i^P + p_i^S)\rangle_0 > 0. \quad (1)$$

In the prisoner's dilemma (the left matrix), the benefit $b > 0$ is gained from the cooperative opponent, and the cost $c > 0$ is paid by the cooperator. In the snowdrift game (the right matrix), the benefit $b > 0$ is gained if at least one side cooperates, and the cost $c > 0$ is divided equally between cooperators.

$$\begin{array}{cc} & C \quad D \\ \begin{array}{c} C \\ D \end{array} & \begin{pmatrix} b-c & -c \\ b & 0 \end{pmatrix} \end{array} \qquad \begin{array}{cc} & C \quad D \\ \begin{array}{c} C \\ D \end{array} & \begin{pmatrix} b-c/2 & b-c \\ b & 0 \end{pmatrix} \end{array}$$

Assuming that an individual interacts with any others in the same group of each layer, the payoff of individual i obtained in the first layer p_i^P and the one in the second p_i^S are as follows. When the two layers use the prisoner's dilemma,

$$p_i^P = \sum_j h_i^P \cdot h_j^P(bs_j^P - cs_i^P) - (b-c)s_i^P,\ \ p_i^S = \sum_j h_i^S \cdot h_j^S(bs_j^S - cs_i^S) - (b-c)s_i^S. \quad (2)$$

The M-dimensional vectors h_i^P and h_i^S, in each of which the k_{th} entry is 1 if individual i is in the k_{th} group and 0 if not, represent the group of individual i in the first and in the second layer, respectively. When the two layers use the snowdrift game,

$$\begin{aligned} p_i^P &= \sum_j h_i^P \cdot h_j^P(b(s_i^P + s_j^P - s_i^P s_j^P) + c(\tfrac{1}{2}s_i^P s_j^P - s_i^P)) - (b - \tfrac{c}{2})s_i^P, \\ p_i^S &= \sum_j h_i^S \cdot h_j^S(b(s_i^S + s_j^S - s_i^S s_j^S) + c(\tfrac{1}{2}s_i^S s_j^S - s_i^S)) - (b - \tfrac{c}{2})s_i^S. \end{aligned} \quad (3)$$

When the first layer uses the prisoner's dilemma and the second the snowdrift game,

$$\begin{aligned} p_i^P &= \sum_j h_i^P \cdot h_j^P(bs_j^P - cs_i^P) - (b-c)s_i^P, \\ p_i^S &= \sum_j h_i^S \cdot h_j^S(b(s_i^S + s_j^S - s_i^S s_j^S) + c(\tfrac{1}{2}s_i^S s_j^S - s_i^S)) - (b - \tfrac{c}{2})s_i^S. \end{aligned} \quad (4)$$

Let $F(s_i^X, h_i^X) = \sum_j h_i^X \cdot h_j^X(bs_j^X - cs_i^X) - (b-c)s_i^X$. When the two layers use the prisoner's dilemma, the elements in Eq. (1) become

$$\begin{aligned} \sum_i s_i^P p_i^P - \sum_{l,i} s_l^P p_i^P/N &= \sum_i s_i^P F(s_i^P, h_i^P) - \sum_{l,i} s_l^P F(s_i^P, h_i^P)/N, \\ \sum_i s_i^P p_i^S - \sum_{l,i} s_l^P p_i^S/N &= \sum_i s_i^P F(s_i^S, h_i^S) - \sum_{l,i} s_l^P F(s_i^S, h_i^S)/N, \\ \sum_i s_i^S p_i^P - \sum_{l,i} s_l^S p_i^P/N &= \sum_i s_i^S F(s_i^P, h_i^P) - \sum_{l,i} s_l^S F(s_i^P, h_i^P)/N, \\ \sum_i s_i^S p_i^S - \sum_{l,i} s_l^S p_i^S/N &= \sum_i s_i^S F(s_i^S, h_i^S) - \sum_{l,i} s_l^S F(s_i^S, h_i^S)/N, \end{aligned} \quad (5)$$

where $\sum_i s_i^X F(s_i^X, h_i^X) - \sum_{l,i} s_l^X F(s_i^X, h_i^X)/N = b(\sum_{i,j} h_i^X \cdot h_j^X s_i^X s_j^X - \sum_i s_i^X - \sum_{l,i,j} h_i^X \cdot h_j^X s_l^X s_j^X/N + \sum_{l,i} s_l^X s_i^X/N) - c(\sum_{i,j} h_i^X \cdot h_j^X s_i^X - \sum_i s_i^X - \sum_{l,i,j} h_i^X \cdot h_j^X s_l^X s_i^X/N + \sum_{l,i} s_l^X s_i^X/N)$ and $\sum_i s_i^X F(s_i^Y, h_i^Y) - \sum_{l,i} s_l^X F(s_i^Y, h_i^Y)/N = b(\sum_{i,j} h_i^Y \cdot h_j^Y s_i^X s_j^Y - \sum_i s_i^X s_i^Y - \sum_{l,i,j} h_i^Y \cdot h_j^Y s_l^X s_j^Y/N + \sum_{l,i} s_l^X s_i^Y/N) - c(\sum_{i,j} h_i^Y \cdot h_j^Y s_i^X s_i^Y - \sum_i s_i^X s_i^Y - \sum_{l,i,j} h_i^Y \cdot h_j^Y s_l^X s_i^Y/N + \sum_{l,i} s_l^X s_i^Y/N)$.

Let $G(s_i^X, h_i^X) = \sum_j h_i^X \cdot h_j^X (b(s_i^X + s_j^X - s_i^X s_j^X) + c(\frac{1}{2} s_i^X s_j^X - s_i^X)) - (b - \frac{c}{2}) s_i^X$. When the two layers use the snowdrift game, the elements in Eq. (1) become

$$\begin{aligned}
\sum_i s_i^P p_i^P - \sum_{l,i} s_l^P p_i^P/N &= \sum_i s_i^P G(s_i^P, h_i^P) - \sum_{l,i} s_l^P G(s_i^P, h_i^P)/N,\\
\sum_i s_i^P p_i^S - \sum_{l,i} s_l^P p_i^S/N &= \sum_i s_i^P G(s_i^S, h_i^S) - \sum_{l,i} s_l^P G(s_i^S, h_i^S)/N,\\
\sum_i s_i^S p_i^P - \sum_{l,i} s_l^S p_i^P/N &= \sum_i s_i^S G(s_i^P, h_i^P) - \sum_{l,i} s_l^S G(s_i^P, h_i^P)/N,\\
\sum_i s_i^S p_i^S - \sum_{l,i} s_l^S p_i^S/N &= \sum_i s_i^S G(s_i^S, h_i^S) - \sum_{l,i} s_l^S G(s_i^S, h_i^S)/N,
\end{aligned} \tag{6}$$

where $\sum_i s_i^X G(s_i^X, h_i^X) - \sum_{l,i} s_l^X G(s_i^X, h_i^X)/N = b(\sum_{i,j} h_i^X \cdot h_j^X s_i^X - \sum_i s_i^X - \sum_{l,i,j} h_i^X \cdot h_j^X (s_l^X s_i^X + s_l^X s_j^X - s_l^X s_i^X s_j^X)/N + \sum_{l,i} s_l^X s_i^X/N) - c(\sum_{i,j} h_i^X \cdot h_j^X (s_i^X - \frac{1}{2} s_i^X s_j^X) - \sum_i s_i^X/2 - \sum_{l,i,j} h_i^X \cdot h_j^X (s_l^X s_i^X - \frac{1}{2} s_l^X s_i^X s_j^X)/N + \sum_{l,i} s_l^X s_i^X/(2N))$ and $\sum_i s_i^X G(s_i^Y, h_i^Y) - \sum_{l,i} s_l^X G(s_i^Y, h_i^Y)/N = b(\sum_{i,j} h_i^Y \cdot h_j^Y (s_i^X s_i^Y + s_i^X s_j^Y - s_i^X s_i^Y s_j^Y) - \sum_i s_i^X s_i^Y - \sum_{l,i,j} h_i^Y \cdot h_j^Y (s_l^X s_i^Y + s_l^X s_j^Y - s_l^X s_i^Y s_j^Y)/N + \sum_{l,i} s_l^X s_i^Y/N) - c(h_i^Y \cdot h_j^Y (s_i^X s_i^Y - \frac{1}{2} s_i^X s_i^Y s_j^Y) - \sum_i s_i^X s_i^Y/2 - \sum_{l,i,j} h_i^Y \cdot h_j^Y (s_l^X s_i^Y - \frac{1}{2} s_l^X s_i^Y s_j^Y)/N + \sum_{l,i} s_l^X s_i^Y/(2N))$.

When the first layer uses the prisoner's dilemma and the second the snowdrift game, the elements in Eq. (1) become

$$\begin{aligned}
\sum_i s_i^P p_i^P - \sum_{l,i} s_l^P p_i^P/N &= \sum_i s_i^P F(s_i^P, h_i^P) - \sum_{l,i} s_l^P F(s_i^P, h_i^P)/N,\\
\sum_i s_i^P p_i^S - \sum_{l,i} s_l^P p_i^S/N &= \sum_i s_i^P G(s_i^S, h_i^S) - \sum_{l,i} s_l^P G(s_i^S, h_i^S)/N,\\
\sum_i s_i^S p_i^P - \sum_{l,i} s_l^S p_i^P/N &= \sum_i s_i^S F(s_i^P, h_i^P) - \sum_{l,i} s_l^S F(s_i^P, h_i^P)/N,\\
\sum_i s_i^S p_i^S - \sum_{l,i} s_l^S p_i^S/N &= \sum_i s_i^S G(s_i^S, h_i^S) - \sum_{l,i} s_l^S G(s_i^S, h_i^S)/N.
\end{aligned} \tag{7}$$

We will show how $\langle \sum_{l,i,j} h_i^P \cdot h_j^P s_l^P s_j^P \rangle_0$ in Eq. (5), (6), and (7) is expressed by the strategy-and-location distribution of multiple individuals in the neutral case. For simplicity, $h^P \cdot h^P$ or $h^S \cdot h^S$ is abbreviated as $h \cdot h$ in the following.

$$\langle \sum_{l,i,j} h_i \cdot h_j s_l^P s_j^P \rangle_0 = N^3 E[\langle h_i \cdot h_j s_l^P s_j^P \rangle_0], \tag{8}$$

where the expectation E is taken over all possible triples of (l, i, j). The sum over l, i, j without limitation means that three individuals are chosen randomly and with replacement from the population: with probability $1/N^2$, all three individuals are identical ($l = i = j$); with probability $(N-1)/N^2$, two given individuals are the same but the third is different ($l = i \neq j$, $l = j \neq i$, or

$i = j \neq l$); with probability $(N-1)(N-2)/N^2$, the three are different from each other ($l \neq i \neq j \neq l$). Therefore,

$$E[\langle h_i \cdot h_j s_l^P s_j^P \rangle_0] = \frac{1}{N^2}\langle s_l^P \rangle_0 + \frac{N-1}{N^2}(\langle h_i \cdot h_j s_i^P s_j^P | i \neq j \rangle_0 + \langle h_i \cdot h_j s_j^P | i \neq j \rangle_0 + \langle s_i^P s_j^P | i \neq j \rangle_0) \\ + \frac{(N-1)(N-2)}{N^2}\langle h_i \cdot h_j s_l^P s_j^P | l \neq i \neq j \neq l \rangle_0. \tag{9}$$

We will make an illustration by the calculation of $\langle h_i \cdot h_j s_l^P s_j^P | l \neq i \neq j \neq l \rangle_0$ in Eq. (9).

$$\begin{aligned} \langle h_i \cdot h_j s_l^P s_j^P | l \neq i \neq j \neq l \rangle_0 &= \langle 1(h_i \cdot h_j = s_l^P = s_j^P = 1 | l \neq i \neq j \neq l) \rangle_0 \\ &= Pr(h_i \cdot h_j = s_l^P = s_j^P = 1 | l \neq i \neq j \neq l), \end{aligned} \tag{10}$$

where the indicator function $1(X)$ is 1 if the event X is true and 0 otherwise, and $Pr(X)$ is the probability that X occurs. The two equal signs in Eq. (10) are self-evident. For simplicity, $Pr(h_i \cdot h_j = s_l^P = s_j^P = 1 | l \neq i \neq j \neq l)$ in Eq. (10) can be understood as the probability that three different individuals labelled by $1, 2, 3$ satisfy $h_2 \cdot h_3 = s_1^P = s_3^P = 1$, and thus is rewritten as $Pr(h_2 \cdot h_3 = s_1^P = s_3^P = 1)$. For intuition, CC, CD, DC, and DD represent $(1,1)$, $(1,0)$, $(0,1)$, and $(0,0)$, respectively. Let $s_1 = (s_1^P, s_1^S)$, $s_2 = (s_2^P, s_2^S)$, $s_3 = (s_3^P, s_3^S)$,

$$\begin{aligned} Pr(h_2 \cdot h_3 = s_1^P = s_3^P = 1) \; = \; & 2Pr(h_2 \cdot h_3 = 1, s_1 = s_2 = s_3 = CC) + 6Pr(h_2 \cdot h_3 = 1, s_1 = s_2 = CC, \\ & s_3 = CD) + 2Pr(h_2 \cdot h_3 = 1, s_1 = s_3 = CC, s_2 = CD) + 2Pr(h_2 \cdot h_3 = 1, \\ & s_2 = s_3 = CC, s_1 = CD) + 4Pr(h_2 \cdot h_3 = 1, s_1 = CC, s_2 = CD, s_3 = DC). \end{aligned} \tag{11}$$

The equal sign in Eq. (11) employs the following equations:

$$\begin{aligned} & Pr(h_2 \cdot h_3 = 1, s_1 = s_2 = s_3 = CC) = Pr(h_2 \cdot h_3 = 1, s_1 = s_2 = s_3 = p), \\ & Pr(h_2 \cdot h_3 = 1, s_1 = s_2 = CC, s_3 = CD) = Pr(h_2 \cdot h_3 = 1, s_1 = s_2 = p, s_3 = q), \\ & Pr(h_2 \cdot h_3 = 1, s_1 = s_3 = CC, s_2 = CD) = Pr(h_2 \cdot h_3 = 1, s_1 = s_3 = p, s_2 = q), \\ & Pr(h_2 \cdot h_3 = 1, s_2 = s_3 = CC, s_1 = CD) = Pr(h_2 \cdot h_3 = 1, s_2 = s_3 = q, s_1 = p), \\ & Pr(h_2 \cdot h_3 = 1, s_1 = CC, s_2 = CD, s_3 = DC) = Pr(h_2 \cdot h_3 = 1, s_1 = p, s_2 = q, s_3 = r), \end{aligned} \tag{12}$$

where $p \neq q \neq r \neq p \in \{CC, CD, DC, DD\}$. These equations hold because all strategies in the neutral stationary state are equivalent, which means $Pr(h_2 \cdot h_3 = 1, s_1 = p, s_2 = q, s_3 = r) = Pr(h_2 \cdot h_3 = 1, s_1 = p', s_2 = q', s_3 = r')$ when a bijection operation from the set $\{CC, CD, DC, DD\}$ to $\{CC, CD, DC, DD\}$ satisfies $\pi((p, q, r)) = (p', q', r')$.

Similar to Eq. (10) and (11), we have

$$\begin{aligned} & \langle s_l^P \rangle_0 = 1/2, \qquad \langle s_i^P s_j^P | i \neq j \rangle_0 = 2Pr(s_1 = s_2 = CC) + 2Pr(s_1 = CC, s_2 = CD), \\ & \langle h_i \cdot h_j s_j^P | i \neq j \rangle_0 = 2Pr(h_1 \cdot h_2 = 1, s_1 = s_2 = CC) + 6Pr(h_1 \cdot h_2 = 1, s_1 = CC, s_2 = CD), \\ & \langle h_i \cdot h_j s_i^P s_j^P | i \neq j \rangle_0 = 2Pr(h_1 \cdot h_2 = 1, s_1 = s_2 = CC) + 2Pr(h_1 \cdot h_2 = 1, s_1 = CC, s_2 = CD). \end{aligned} \tag{13}$$

Finally based on Eq. (8), (9), (10), (11), and (13), we arrive at

$$
\begin{aligned}
\langle \textstyle\sum_{l,i,j} h_i \cdot h_j s_l^P s_i^P \rangle_0 = \tfrac{N}{2} + N(N-1)(2Pr(s_1 = s_2 = CC) + 2Pr(s_1 = CC, s_2 = CD) + 4Pr(h_1 \cdot h_2 = 1, \\
s_1 = s_2 = CC) + 8Pr(h_1 \cdot h_2 = 1, s_1 = CC, s_2 = CD)) + N(N-1)(N-2)(2Pr(h_2 \cdot h_3 = 1, s_1 = s_2 \\
= s_3 = CC) + 6Pr(h_2 \cdot h_3 = 1, s_1 = s_2 = CC, s_3 = CD) + 2Pr(h_2 \cdot h_3 = 1, s_1 = s_3 = CC, s_2 = CD) \\
+2Pr(h_2 \cdot h_3 = 1, s_2 = s_3 = CC, s_1 = CD,) + 4Pr(h_2 \cdot h_3 = 1, s_1 = CC, s_2 = CD, s_3 = DC)).
\end{aligned} \tag{14}
$$

Similar to Eq. (14), we can get Eq. (15) and (16):

$$
\begin{aligned}
&\langle \textstyle\sum_{i,j} h_i \cdot h_j s_i^P s_j^S \rangle_0 = \langle \textstyle\sum_{i,j} h_i \cdot h_j s_i^S s_j^P \rangle_0 = \tfrac{N}{4} + N(N-1)(Pr(h_1 \cdot h_2 = 1, s_1 = s_2 = CC) \\
&\quad +3Pr(h_1 \cdot h_2 = 1, s_1 = CC, s_2 = CD)), \\
&\langle \textstyle\sum_{i,j} h_i \cdot h_j s_i^P s_i^S \rangle_0 = \tfrac{N}{4} + N(N-1)(Pr(h_1 \cdot h_2 = 1, s_1 = s_2 = CC) + 3Pr(h_1 \cdot h_2 = 1, s_1 = CC, s_2 = CD)), \\
&\langle \textstyle\sum_{i,j} h_i \cdot h_j s_i^P s_i^S s_j^S \rangle_0 = \tfrac{N}{4} + N(N-1)(Pr(h_1 \cdot h_2 = 1, s_1 = s_2 = CC) + Pr(h_1 \cdot h_2 = 1, s_1 = CC, s_2 = CD)), \\
&\langle \textstyle\sum_{l,i,j} h_i \cdot h_j s_l^S s_i^P \rangle_0 = \langle \textstyle\sum_{l,i,j} h_i \cdot h_j s_l^S s_j^P \rangle_0 = \langle \textstyle\sum_{l,i,j} h_i \cdot h_j s_l^P s_j^S \rangle_0 = \langle \textstyle\sum_{l,i,j} h_i \cdot h_j s_l^P s_i^S \rangle_0 = \\
&\quad \tfrac{N}{4} + N(N-1)(Pr(s_1 = s_2 = CC) + 3Pr(s_1 = CC, s_2 = CD) + 2Pr(h_1 \cdot h_2 = 1, s_1 = s_2 = CC) \\
&\quad +6Pr(h_1 \cdot h_2 = 1, s_1 = CC, s_2 = CD)) + N(N-1)(N-2)(Pr(h_2 \cdot h_3 = 1, s_1 = s_2 = s_3 = CC) \\
&\quad +3Pr(h_2 \cdot h_3 = 1, s_1 = s_2 = CC, s_3 = CD) + 3Pr(h_2 \cdot h_3 = 1, s_1 = s_3 = CC, s_2 = CD) \\
&\quad +3Pr(h_2 \cdot h_3 = 1, s_2 = s_3 = CC, s_1 = CD) + 6Pr(h_2 \cdot h_3 = 1, s_1 = CC, s_2 = CD, s_3 = DC)), \\
&\langle \textstyle\sum_{l,i,j} h_i \cdot h_j s_l^P s_i^S s_j^S \rangle_0 = \tfrac{N}{4} + N(N-1)(Pr(s_1 = s_2 = CC) + 3Pr(s_1 = CC, s_2 = CD) \\
&\quad +2Pr(h_1 \cdot h_2 = 1, s_1 = s_2 = CC) + 2Pr(h_1 \cdot h_2 = 1, s_1 = CC, s_2 = CD)) + N(N-1)(N-2) \\
&\quad (Pr(h_2 \cdot h_3 = 1, s_1 = s_2 = s_3 = CC) + Pr(h_2 \cdot h_3 = 1, s_1 = s_2 = CC, s_3 = CD) \\
&\quad +Pr(h_2 \cdot h_3 = 1, s_1 = s_3 = CC, s_2 = CD) + 3Pr(h_2 \cdot h_3 = 1, s_2 = s_3 = CC, s_1 = CD) \\
&\quad +2Pr(h_2 \cdot h_3 = 1, s_1 = CC, s_2 = CD, s_3 = DC)).
\end{aligned} \tag{15}
$$

$$
\begin{aligned}
&\langle\textstyle\sum_{i,j} h_i\cdot h_j s_i^P s_j^P\rangle_0 = \langle\sum_{i,j} h_i\cdot h_j s_i^S s_j^S\rangle_0 = \frac{N}{2} + N(N-1)(2Pr(h_1\cdot h_2=1, s_1=s_2=CC)\\
&\qquad +2Pr(h_1\cdot h_2=1, s_1=CC, s_2=CD)),\\
&\langle\textstyle\sum_{i,j} h_i\cdot h_j s_i^P\rangle_0 = \langle\sum_{i,j} h_i\cdot h_j s_i^S\rangle_0 = \frac{N}{2} + N(N-1)(2Pr(h_1\cdot h_2=1, s_1=s_2=CC)\\
&\qquad +6Pr(h_1\cdot h_2=1, s_1=CC, s_2=CD)),\\
&\langle\textstyle\sum_{l,i,j} h_i\cdot h_j s_l^P s_i^P\rangle_0 = \langle\sum_{l,i,j} h_i\cdot h_j s_l^P s_j^P\rangle_0 = \langle\sum_{l,i,j} h_i\cdot h_j s_l^S s_j^S\rangle_0 = \langle\sum_{l,i,j} h_i\cdot h_j s_l^S s_i^S\rangle_0 =\\
&\qquad \frac{N}{2} + N(N-1)(2Pr(s_1=s_2=CC) + 2Pr(s_1=CC, s_2=CD) + 4Pr(h_1\cdot h_2=1, s_1=s_2=CC)\\
&\qquad +8Pr(h_1\cdot h_2=1, s_1=CC, s_2=CD)) + N(N-1)(N-2)(2Pr(h_2\cdot h_3=1, s_1=s_2=s_3=CC)\\
&\qquad +6Pr(h_2\cdot h_3=1, s_1=s_2=CC, s_3=CD) + 2Pr(h_2\cdot h_3=1, s_1=s_3=CC, s_2=CD)\\
&\qquad +2Pr(h_2\cdot h_3=1, s_2=s_3=CC, s_1=CD) + 4Pr(h_2\cdot h_3=1, s_1=CC, s_2=CD, s_3=DC)),\\
&\langle\textstyle\sum_{l,i,j} h_i\cdot h_j s_l^S s_i^S s_j^S\rangle_0 = \frac{N}{2} + N(N-1)(2Pr(s_1=s_2=CC) + 2Pr(s_1=CC, s_2=CD)\\
&\qquad +4Pr(h_1\cdot h_2=1, s_1=s_2=CC) + 4Pr(h_1\cdot h_2=1, s_1=CC, s_2=CD)) + N(N-1)(N-2)\\
&\qquad (2Pr(h_2\cdot h_3=1, s_1=s_2=s_3=CC) + 2Pr(h_2\cdot h_3=1, s_1=s_2=CC, s_3=CD)\\
&\qquad +2Pr(h_2\cdot h_3=1, s_1=s_3=CC, s_2=CD) + 2Pr(h_2\cdot h_3=1, s_2=s_3=CC, s_1=CD)).
\end{aligned}
\tag{16}
$$

Based on Eq. (15), we have $\langle\sum_i s_i^S p_i^P - \frac{1}{N}\sum_{l,i} s_l^S p_i^P\rangle_0 = 0$ and $\langle\sum_i s_i^P p_i^S - \frac{1}{N}\sum_{l,i} s_l^P p_i^S\rangle_0 = 0$. Accordingly, the condition for cooperation in Eq. (1) becomes

$$
\omega\langle\textstyle\sum_i s_i^P p_i^P - \frac{1}{N}\sum_{l,i} s_l^P p_i^P\rangle_0 + (1-\omega)\langle\sum_i s_i^S p_i^S - \frac{1}{N}\sum_{l,i} s_l^S p_i^S\rangle_0 > 0. \tag{17}
$$

Based on Eq. (16) and integrating the probabilities, we get the following conclusions. When the two layers use the prisoner's dilemma,

$$
\begin{aligned}
&\omega\langle\textstyle\sum_i s_i^P p_i^P - \frac{1}{N}\sum_{l,i} s_l^P p_i^P\rangle_0 + (1-\omega)\langle\sum_i s_i^S p_i^S - \frac{1}{N}\sum_{l,i} s_l^S p_i^S\rangle_0\\
&= \frac{N-1}{3} b(\omega(-\beta^P + (N-1)\gamma^P - (N-2)\eta^P) + (1-\omega)(-\beta^S + (N-1)\gamma^S - (N-2)\eta^S))\\
&-\frac{N-1}{3} c(\omega((N-1)\beta^P - \gamma^P - (N-2)\eta^P) + (1-\omega)((N-1)\beta^S - \gamma^S - (N-2)\eta^S)).
\end{aligned}
\tag{18}
$$

The above β^P, γ^P, η^P, β^S, γ^S, and η^S describe the strategy-and-location distribution of multiple individuals in the neutral selection: when two individuals are chosen without replacement, β^P (β^S) is the chance that they reside in one group of the first layer (of the second), and γ^P (γ^S) is the probability that they have both the same strategies in the two layers and the same group of the first layer (of the second); when three individuals are chosen without replacement, η^P (η^S) is the probability that the former two have the same strategies in the two layers and the latter two are in

one group of the first layer (of the second). When the two layers use the snowdrift game,

$$
\begin{aligned}
&\omega\langle \textstyle\sum_i s_i^P p_i^P - \frac{1}{N}\sum_{l,i} s_l^P p_i^P\rangle_0 + (1-\omega)\langle \sum_i s_i^S p_i^S - \frac{1}{N}\sum_{l,i} s_l^S p_i^S\rangle_0 \\
&= \tfrac{N-1}{3} b(\omega(N-2)(\tfrac{1}{2}\beta^P + \tfrac{1}{2}\gamma^P - \eta^P) + (1-\omega)(N-2)(\tfrac{1}{2}\beta^S + \tfrac{1}{2}\gamma^S - \eta^S)) \\
&-\tfrac{N-1}{3} c(\omega(\tfrac{3N-2}{4}\beta^P - \tfrac{N+2}{4}\gamma^P - \tfrac{N-2}{2}\eta^P) + (1-\omega)(\tfrac{3N-2}{4}\beta^S - \tfrac{N+2}{4}\gamma^S - \tfrac{N-2}{2}\eta^S)).
\end{aligned} \tag{19}
$$

When the first layer uses the prisoner's dilemma and the second the snowdrift game,

$$
\begin{aligned}
&\omega\langle \textstyle\sum_i s_i^P p_i^P - \frac{1}{N}\sum_{l,i} s_l^P p_i^P\rangle_0 + (1-\omega)\langle \sum_i s_i^S p_i^S - \frac{1}{N}\sum_{l,i} s_l^S p_i^S\rangle_0 \\
&= \tfrac{N-1}{3} b(\omega(-\beta^P + (N-1)\gamma^P - (N-2)\eta^P) + (1-\omega)(N-2)(\tfrac{1}{2}\beta^S + \tfrac{1}{2}\gamma^S - \eta^S)) \\
&-\tfrac{N-1}{3} c(\omega((N-1)\beta^P - \gamma^P - (N-2)\eta^P) + (1-\omega)(\tfrac{3N-2}{4}\beta^S - \tfrac{N+2}{4}\gamma^S - \tfrac{N-2}{2}\eta^S)).
\end{aligned} \tag{20}
$$

II. A METHOD COMBINING THE COALESCENCE THEORY WITH THE THEORY OF RANDOM WALKS

In each update, there is a single newborn offspring (labelled as O) who was reproduced and a single parent (labelled as P) who reproduced in the immediately previous generation. Two possibilities occur upon the whole population in each update: with probability $1/N$, the individual chosen to reproduce is identical to the one chosen to die; with the remaining probability, they are different. In the first case, three cases happen to the O and the P: there is no chance that they coexist in the sample; with probability $\frac{1}{N}$, I_x of the sample is the O and none of the sample are the P; with probability $\frac{N-k}{N}$, there is not the O in the sample. In the second case, three cases still happen to the O and the P: with probability $\frac{1}{N(N-1)}$, I_x and I_y of the sample are the O and the P, respectively; with probability $\frac{N-k}{N(N-1)}$, I_x of the sample is the O and the rest are not the P; with probability $\frac{N-k}{N}$, the O does not appear in the sample. Combining these two cases, we have the following conclusions: with probability $\frac{1}{N^2}$, I_x and I_y of the sample are the O and the P, respectively; with probability $\frac{N-k+1}{N^2}$, I_x of the sample is the O and the rest are not the P; with probability $\frac{N-k}{N}$, none of the sample are the O.

III. THE CALCULATION OF $\beta^P, \gamma^P, \beta^S$ AND γ^S

In the following, let s_i and m_i be the strategy and the location of individual i. Obviously, the probability that one individual is located in η_1 ($\in \{1, \cdots, M\}^2$) and uses the strategy θ_1 ($\in$

$\{1, \cdots, S\}$) $P(s_1 = \theta_1; m_1 = \eta_1)$ is

$$P(s_1 = \theta_1; m_1 = \eta_1) = \frac{1}{M^2 S}. \tag{21}$$

Based on Eq. (21), we can obtain the probability that two different individuals are located in $\gamma_1, \gamma_2 (\in \{1, \cdots, M\}^2)$ respectively and use strategies $\delta_1, \delta_2 (\in \{1, \cdots, S\})$ respectively $P(s_1 = \delta_1, s_2 = \delta_2; m_1 = \gamma_1, m_2 = \gamma_2)$:

$$\begin{aligned}
&P(s_1 = \delta_1, s_2 = \delta_2; m_1 = \gamma_1, m_2 = \gamma_2)\\
&= \sum_{\theta_1,\eta_1} P(s_1 = \theta_1; m_1 = \eta_1) \sum_{T=1}^{+\infty} \frac{1}{N^2} (\sum_{k_1=1}^{T} \sum_{k_2=0}^{T-k_1} P(k_1 - 1, k_2) + \sum_{k_1=0}^{T-1} \sum_{k_2=1}^{T-k_1} P(k_1, k_2 - 1))\\
&\sum_{g_1,h_1=0}^{k_1} \sum_{g_2,h_2=0}^{k_2} M(g_1, g_2; h_1, h_2) Pr_M(\eta_1 \xrightarrow{g_1} \gamma_1) Pr_M(\eta_1 \xrightarrow{g_2} \gamma_2) Pr_S(\theta_1 \xrightarrow{h_1} \delta_1) Pr_S(\theta_1 \xrightarrow{h_2} \delta_2)\\
&= \frac{1}{M^4 S^2} \sum_{x_1+x_2=M\times 1_2 \,\mathrm{or}\, 2M\times 1_2} \sum_{y_1+y_2=S \,\mathrm{or}\, 2S} \Psi(f(x_1), f(x_2), g(y_1), g(y_2)) \exp\{-\frac{2\pi\iota}{M}(x_1 \cdot \gamma_1\\
&+x_2 \cdot \gamma_2)\} \exp\{-\frac{2\pi\iota}{S}(y_1 \cdot \delta_1 + y_2 \cdot \delta_2)\},
\end{aligned} \tag{22}$$

where

$$\Psi(f(x_1), f(x_2), g(y_1), g(y_2)) = \frac{(1-u)(1-v)+(1-u)v\sum_{i=1}^{2} f(x_i)/2+(1-v)u\sum_{i=1}^{2} g(y_i)/2+uv\sum_{i=1}^{2} f(x_i)g(y_i)/2}{1+(N-1)(1-u)v(1-\sum_{i=1}^{2}\frac{f(x_i)}{2})+(N-1)(1-v)u(1-\sum_{i=1}^{2}\frac{g(y_i)}{2})+(N-1)uv(1-\sum_{i=1}^{2}\frac{f(x_i)g(y_i)}{2})} \tag{23}$$

1_2 is the two-dimensional vector with all elements as 1.

For intuition, let $\gamma_1 = (\gamma_{11}, \gamma_{12})$, $\gamma_2 = (\gamma_{21}, \gamma_{22})$, $x_1 = (x_{11}, x_{12})$, and $x_2 = (x_{21}, x_{22})$. The probability that two different individuals are located in the same group of the first layer β^P is

$$\begin{aligned}
\beta^P &= \sum_{\delta_1,\delta_2=1}^{S} \sum_{\gamma_{11}=\gamma_{21}=1}^{M} \sum_{\gamma_{12},\gamma_{22}=1}^{M} P(s_1 = \delta_1, s_2 = \delta_2; m_1 = (\gamma_{11}, \gamma_{12}), m_2 = (\gamma_{21}, \gamma_{22}))\\
&= \frac{1}{M} \sum_{y_1=y_2=S} \sum_{x_{11}+x_{21}=M \,\mathrm{or}\, 2M} \sum_{x_{12}=x_{22}=M} \Psi(f(x_{11}, x_{12}), f(x_{21}, x_{22}), g(y_1), g(y_2)).
\end{aligned} \tag{24}$$

The second equal sign in Eq. (24) holds because $\sum_{\delta_1,\delta_2=1}^{S}$, $\sum_{\gamma_{11}=\gamma_{21}=1}^{M}$, and $\sum_{\gamma_{12},\gamma_{22}=1}^{M}$ mean $\Psi(f(x_{11}, x_{12}), f(x_{21}, x_{22}), g(y_1), g(y_2))$ does not vanish only if $y_1 = y_2 = S$, only if $x_{11} + x_{21} = M \,\mathrm{or}\, 2M$, and only if $x_{12} = x_{22} = M$, respectively. In our model, the mutated offspring adopts one of the strategy space equi-probably, then the corresponding $g(r)$ is $g(r) = \frac{1}{S}(1 + \cos\frac{2\pi r}{S} + \cdots + \cos\frac{2\pi(S-1)r}{S})$ and satisfies

$$\begin{aligned}
&g(y_1) = g(y_2) = 1, && \text{when}\, y_1 = y_2 = S;\\
&g(y_1) = g(y_2) = 0, && \text{when}\, y_1 + y_2 = S;\\
&f(x_{11}, x_{12}) = f(x_{21}, x_{22}) = f(x_{11}, M), && \text{when}\, x_{11} + x_{21} = M \,\mathrm{or}\, 2M, x_{12} = x_{22} = M.
\end{aligned} \tag{25}$$

Taking Eq. (25) into (23) and (24),

$$\beta^P = \frac{1}{M} \sum_{x_{11}=1}^{M} \Psi_1(f(x_{11}, M)), \tag{26}$$

where $\Psi_1(f) = \frac{1-v+vf}{1+(N-1)v(1-f)}$. Following the example of the analysis of Eq. (26), the probability that two different individuals are located in the same group of the second layer β^S is

$$\begin{aligned}\beta^S &= \sum_{\delta_1,\delta_2=1}^{S} \sum_{\gamma_{11},\gamma_{21}=1}^{M} \sum_{\gamma_{12}=\gamma_{22}=1}^{M} P(s_1=\delta_1, s_2=\delta_2; m_1=(\gamma_{11},\gamma_{12}), m_2=(\gamma_{21},\gamma_{22})) \\ &= \frac{1}{M} \sum_{y_1=y_2=S} \sum_{x_{11}=x_{21}=M} \sum_{x_{12}+x_{22}=M \,\mathrm{or}\, 2M} \Psi(f(x_{11},x_{12}), f(x_{21},x_{22}), g(y_1), g(y_2)) \\ &= \frac{1}{M} \sum_{x_{12}=1}^{M} \Psi_1(f(M,x_{12})),\end{aligned} \tag{27}$$

the probability that two different individuals have both the same strategies in the two layers and the same group in the first γ^P is

$$\begin{aligned}\gamma^P &= \sum_{\delta_1=\delta_2=1}^{S} \sum_{\gamma_{11}=\gamma_{21}=1}^{M} \sum_{\gamma_{12},\gamma_{22}=1}^{M} P(s_1=\delta_1, s_2=\delta_2; m_1=(\gamma_{11},\gamma_{12}), m_2=(\gamma_{21},\gamma_{22})) \\ &= \frac{1}{MS} \sum_{y_1+y_2=S \,\mathrm{or}\, 2S} \sum_{x_{11}+x_{21}=M \,\mathrm{or}\, 2M} \sum_{x_{12}=x_{22}=M} \Psi(f(x_{11},x_{12}), f(x_{21},x_{22}), g(y_1), g(y_2)) \\ &= \frac{1}{MS} \sum_{x_{11}=1}^{M} (\Psi_1(f(x_{11},M)) + (S-1)\Psi_2(f(x_{11},M))),\end{aligned} \tag{28}$$

where $\Psi_2(f) = \frac{(1-u)(1-v+vf)}{1+(N-1)u+(N-1)(1-u)v(1-f)}$, the probability that two different individuals have both the same strategies in the two layers and the same group in the second γ^S is

$$\begin{aligned}\gamma^S &= \sum_{\delta_1=\delta_2=1}^{S} \sum_{\gamma_{11},\gamma_{21}=1}^{M} \sum_{\gamma_{12}=\gamma_{22}=1}^{M} P(s_1=\delta_1, s_2=\delta_2; m_1=(\gamma_{11},\gamma_{12}), m_2=(\gamma_{21},\gamma_{22})) \\ &= \frac{1}{MS} \sum_{y_1+y_2=S \,\mathrm{or}\, 2S} \sum_{x_{11}=x_{21}=M} \sum_{x_{12}+x_{22}=M \,\mathrm{or}\, 2M} \Psi(f(x_{11},x_{12}), f(x_{21},x_{22}), g(y_1), g(y_2)) \\ &= \frac{1}{MS} \sum_{x_{12}=1}^{M} (\Psi_1(f(M,x_{12})) + (S-1)\Psi_2(f(M,x_{12}))).\end{aligned} \tag{29}$$

IV. THE CALCULATION η^S AND η^S

Based on Eq. (22), we can obtain the probability that three different individuals are located in $\gamma_1, \gamma_2, \gamma_3\,(\in \{1,\cdots,M\}^2)$ respectively and use strategies $\delta_1, \delta_2, \delta_3\,(\in \{1,\cdots,S\})$ respectively

$P(s_1=\delta_1, s_2=\delta_2, s_3=\delta_3; m_1=\gamma_1, m_2=\gamma_2, m_3=\gamma_3)$:

$$
\begin{aligned}
&P(s_1=\delta_1, s_2=\delta_2, s_3=\delta_3; m_1=\gamma_1, m_2=\gamma_2, m_3=\gamma_3)\\
&=\sum_{\theta_1,\theta_2,\eta_1,\eta_2} P(s_1=\theta_1, s_2=\theta_2; m_1=\eta_1, m_2=\eta_2)\sum_{T=1}^{+\infty}\frac{1}{N^2}\{(\sum_{k_1=1}^{T}\sum_{k_2=0}^{T-k_1}\sum_{k_3=0}^{T-k_1-k_2} P(k_1-1,\\
&k_2,k_3)+\sum_{k_1=0}^{T-1}\sum_{k_2=1}^{T-k_1}\sum_{k_3=0}^{T-k_1-k_2} P(k_1,k_2-1,k_3))\sum_{g_1,h_1=0}^{k_1}\sum_{g_2,h_2=0}^{k_2}\sum_{g_3,h_3=0}^{k_3} M(g_1,g_2,g_3;h_1,h_2,\\
&h_3)Pr_M(\eta_1\xrightarrow{g_1}\gamma_1)Pr_M(\eta_1\xrightarrow{g_2}\gamma_2)\,Pr_S(\theta_1\xrightarrow{h_1}\delta_1)\,Pr_S(\theta_1\xrightarrow{h_2}\delta_2)Pr_M(\eta_2\xrightarrow{g_3}\gamma_3)Pr_S(\theta_2\xrightarrow{h_3}\delta_3)\\
&+(\sum_{k_1=1}^{T}\sum_{k_2=0}^{T-k_1}\sum_{k_3=0}^{T-k_1-k_2} P(k_1-1,k_2,k_3)+\sum_{k_1=0}^{T-1}\sum_{k_2=0}^{T-k_1-1}\sum_{k_3=1}^{T-k_1-k_2} P(k_1,k_2,k_3-1))\sum_{g_1,h_1=0}^{k_1}\\
&\sum_{g_2,h_2=0}^{k_2}\sum_{g_3,h_3=0}^{k_3} M(g_1,g_2,g_3;h_1,h_2,h_3)Pr_M(\eta_1\xrightarrow{g_1}\gamma_1)Pr_M(\eta_1\xrightarrow{g_3}\gamma_3)Pr_S(\theta_1\xrightarrow{h_1}\delta_1)Pr_S(\theta_1\\
&\xrightarrow{h_3}\delta_3)Pr_M(\eta_2\xrightarrow{g_2}\gamma_2)Pr_S(\theta_2\xrightarrow{h_2}\delta_2)+(\sum_{k_1=0}^{T-1}\sum_{k_2=1}^{T-k_1}\sum_{k_3=0}^{T-k_1-k_2} P(k_1,k_2-1,k_3)+\sum_{k_1=0}^{T-1}\sum_{k_2=0}^{T-k_1-1}\\
&\sum_{k_3=1}^{T-k_1-k_2} P(k_1,k_2,k_3-1))\sum_{g_1,h_1=0}^{k_1}\sum_{g_2,h_2=0}^{k_2}\sum_{g_3,h_3=0}^{k_3} M(g_1,g_2,g_3;h_1,h_2,h_3)Pr_M(\eta_1\xrightarrow{g_2}\gamma_2)\\
&Pr_M(\eta_1\xrightarrow{g_3}\gamma_3)Pr_S(\theta_1\xrightarrow{h_2}\delta_2)Pr_S(\theta_1\xrightarrow{h_3}\delta_3)Pr_M(\eta_2\xrightarrow{g_1}\gamma_1)Pr_S(\theta_2\xrightarrow{h_1}\delta_1)\}\\
&=\frac{1}{3M^6S^3}\sum_{z_1+z_2+z_3=M\times 1_2,2M\times 1_2 or 3M\times 1_2}\sum_{w_1+w_2+w_3=S,2S or 3S}\quad\{\sum_{x_1=z_1+z_2,x_2=z_3,y_1=w_1+w_2,y_2=w_3}+\\
&\sum_{x_1=z_1+z_3,x_2=z_2,y_1=w_1+w_3,y_2=w_2}+\sum_{x_1=z_2+z_3,x_2=z_1,y_1=w_2+w_3,y_2=w_1}\}\Phi(f(z_1),f(z_2),f(z_3),\\
&g(w_1),g(w_2),g(w_3))\times\Psi(f(x_1),f(x_2),g(y_1),g(y_2))\exp\{-\tfrac{2\pi\iota}{M}(z_1\cdot\gamma_1+z_2\cdot\gamma_2+z_3\cdot\gamma_3]\}\\
&\exp\{-\tfrac{2\pi\iota}{S}[w_1\cdot\delta_1+w_2\cdot\delta_2+w_3\cdot\delta_3]\},
\end{aligned}
\tag{30}
$$

where

$$
\Phi(f(z_1),f(z_2),f(z_3),g(w_1),g(w_2),g(w_3))=
\begin{cases}
\frac{2(1-u)(1-v)+(1-u)v(f(z_1)+f(z_2))+(1-v)u(g(w_1)+g(w_2))+uv(f(z_1)g(w_1)+f(z_2)g(w_2))}{2+(N-2)(1-u)v(1-\sum_{i=1}^3 f(z_i)/3)+(N-2)(1-v)u(1-\sum_{i=1}^3 g(w_i)/3)+(N-2)uv(1-\sum_{i=1}^3 f(z_i)g(w_i)/3)},\\
\quad\text{if } x_1=z_1+z_2, x_2=z_3, y_1=w_1+w_2, y_2=w_3;\\
\frac{2(1-u)(1-v)+(1-u)v(f(z_1)+f(z_3))+(1-v)u(g(w_1)+g(w_3))+uv(f(z_1)g(w_1)+f(z_3)g(w_3))}{2+(N-2)(1-u)v(1-\sum_{i=1}^3 f(z_i)/3)+(N-2)(1-v)u(1-\sum_{i=1}^3 g(w_i)/3)+(N-2)uv(1-\sum_{i=1}^3 f(z_i)g(w_i)/3)},\\
\quad\text{if } x_1=z_1+z_3, x_2=z_2, y_1=w_1+w_3, y_2=w_2;\\
\frac{2(1-u)(1-v)+(1-u)v(f(z_2)+f(z_3))+(1-v)u(g(w_2)+g(w_3))+uv(f(z_2)g(w_2)+f(z_3)g(w_3))}{2+(N-2)(1-u)v(1-\sum_{i=1}^3 f(z_i)/3)+(N-2)(1-v)u(1-\sum_{i=1}^3 g(w_i)/3)+(N-2)uv(1-\sum_{i=1}^3 f(z_i)g(w_i)/3)},\\
\quad\text{if } x_1=z_2+z_3, x_2=z_1, y_1=w_2+w_3, y_2=w_1.
\end{cases}
\tag{31}
$$

For intuition, let $\gamma_1=(\gamma_{11},\gamma_{12})$, $\gamma_2=(\gamma_{21},\gamma_{22})$, $\gamma_3=(\gamma_{31},\gamma_{32})$, $z_1=(z_{11},z_{12})$, $z_2=(z_{21},z_{22})$, and $z_3=(z_{31},z_{32})$. Following the example of the analysis of Eq. (24), the probability that the former two of three different individuals have the same strategies in the two layers and the latter two are located in the same group of the first layer η^P is

$$
\begin{aligned}
\eta^P &= \sum_{\delta_1=\delta_2=1}^{S}\sum_{\delta_3=1}^{S}\sum_{\gamma_{11}=1}^{M}\sum_{\gamma_{21}=\gamma_{31}=1}^{M}\sum_{\gamma_{12}=\gamma_{22}=\gamma_{32}=M} P(s_1=\delta_1,s_2=\delta_2,s_3=\delta_3;m_1=(\gamma_{11},\gamma_{12}),\\
&\quad m_2=(\gamma_{21},\gamma_{22}),m_3=(\gamma_{31},\gamma_{32}))\\
&= \frac{1}{3MS}\sum_{z_{11}=M,z_{21}+z_{31}=M\text{ or }2M}\sum_{z_{12}=z_{22}=z_{32}=M}\sum_{w_1+w_2=S\text{ or }2S,w_3=S}\{\sum_{x_1=z_1+z_2,x_2=z_3,y_1=w_1+w_2,y_2=w_3}\\
&\quad+\sum_{x_1=z_1+z_3,x_2=z_2,y_1=w_1+w_3,y_2=w_2}+\sum_{x_1=z_2+z_3,x_2=z_1,y_1=w_2+w_3,y_2=w_1}\}\Phi(f(z_{11},z_{12}),f(z_{21},z_{22}),\\
&\quad f(z_{31},z_{32}),g(w_1),g(w_2),g(w_3))\times\Psi(f(x_{11},x_{12}),f(x_{21},x_{22}),g(y_1),g(y_2)).
\end{aligned}
\tag{32}
$$

When $x_1 = z_1 + z_2, x_2 = z_3, y_1 = w_1 + w_2, y_2 = w_3$,

$$\begin{cases} f(z_{11}, z_{12}) = 1, f(z_{21}, z_{22}) = f(z_{31}, z_{32}) = f(z_{21}, M), f(x_{11}, x_{12}) = f(x_{21}, x_{22}) \\ = f(z_{21}, M), \quad \text{when } z_{11} = M, z_{21} + z_{31} = M \text{ or } 2M, z_{12} = z_{22} = z_{32} = M\,; \\ g(w_1) = g(w_2) = 0, g(w_3) = 1, g(y_1) = g(y_2) = 1, \quad \text{when } w_1 + w_2 = S, w_3 = S\,; \\ g(w_1) = g(w_2) = g(w_3) = g(y_1) = g(y_2) = 1, \quad \text{when } w_1 = w_2 = w_3 = S. \end{cases} \tag{33}$$

When $x_1 = z_1 + z_3, x_2 = z_2, y_1 = w_1 + w_3, y_2 = w_2$,

$$\begin{cases} f(z_{11}, z_{12}) = 1, f(z_{21}, z_{22}) = f(z_{31}, z_{32}) = f(z_{21}, M), f(x_{11}, x_{12}) = f(x_{21}, x_{22}) \\ = f(z_{21}, M), \quad \text{when } z_{11} = M, z_{21} + z_{31} = M \text{ or } 2M, z_{12} = z_{22} = z_{32} = M\,; \\ g(w_1) = g(w_2) = 0, g(w_3) = 1, g(y_1) = g(y_2) = 0, \quad \text{when } w_1 + w_2 = S, w_3 = S\,; \\ g(w_1) = g(w_2) = g(w_3) = g(y_1) = g(y_2) = 1, \quad \text{when } w_1 = w_2 = w_3 = S. \end{cases} \tag{34}$$

When $x_1 = z_2 + z_3, x_2 = z_1, y_1 = w_2 + w_3, y_2 = w_1$,

$$\begin{cases} f(z_{11}, z_{12}) = 1, f(z_{21}, z_{22}) = f(z_{31}, z_{32}) = f(z_{21}, M), f(x_{11}, x_{12}) = f(x_{21}, x_{22}) \\ = 1, \quad \text{when } z_{11} = M, z_{21} + z_{31} = M \text{ or } 2M, z_{12} = z_{22} = z_{32} = M\,; \\ g(w_1) = g(w_2) = 0, g(w_3) = 1, g(y_1) = g(y_2) = 0, \quad \text{when } w_1 + w_2 = S, w_3 = S\,; \\ g(w_1) = g(w_2) = g(w_3) = g(y_1) = g(y_2) = 1, \quad \text{when } w_1 = w_2 = w_3 = S. \end{cases} \tag{35}$$

Taking Eq. (33), (34), and (35) into Eq. (31) and (23), the expressions of $\Phi(f(z_{11}, z_{12}), f(z_{21}, z_{22}), f(z_{31}, z_{32}), g(w_1), g(w_2), g(w_3)) \times \Psi(f(x_{11}, x_{12}), f(x_{21}, x_{22}), g(y_1), g(y_2))$ over the points satisfying $w_1 = w_2 = w_3 = S$ or $w_1 + w_2 = S, w_3 = S$ are summed up as Table 1. Finally by using $\Phi_4 + 2\Phi_5\Psi_1 = 3\Psi_1$, we arrive at

$$\begin{aligned} \eta^P = \ & \tfrac{1}{3MS} \textstyle\sum_{x_{21}=1}^{M} (3\Psi_1(f(x_{21}, M)) + (S-1)(\Phi_1(f(x_{21}, M))\alpha_1 + \Phi_2(f(x_{21}, M))\Psi_1(f(x_{21}, M)) \\ & + \Phi_3(f(x_{21}, M))\Psi_2(f(x_{21}, M))). \end{aligned} \tag{36}$$

Similarly, the probability that the former two of three different individuals have the same strategies in the two layers and the latter two are located in the same group of the second layer η^S is

$$\begin{aligned} \eta^S = \ & \textstyle\sum_{\delta_1=\delta_2=1}^{S} \sum_{\delta_3=1}^{S} \sum_{\gamma_{11},\gamma_{21},\gamma_{31}=1}^{M} \sum_{\gamma_{12}=1}^{M} \sum_{\gamma_{22}=\gamma_{32}=1}^{M} P(s_1 = \delta_1, s_2 = \delta_2, s_3 = \delta_3; \\ & m_1 = (\gamma_{11}, \gamma_{12}), m_2 = (\gamma_{21}, \gamma_{22}), m_3 = (\gamma_{31}, \gamma_{32})) \\ = \ & \tfrac{1}{3MS} \textstyle\sum_{x_{22}=1}^{M} (3\Psi_1(f(M, x_{22})) + (S-1)(\Phi_1(f(M, x_{22}))\alpha_1 + \Phi_2(f(M, x_{22}))\Psi_1(f(M, x_{22})) \\ & + \Phi_3(f(M, x_{22}))\Psi_2(f(M, x_{22}))). \end{aligned} \tag{37}$$

TABLE I: The expressions of $\Phi(f(z_{11}, z_{12}), f(z_{21}, z_{22}), f(z_{31}, z_{32}), g(w_1), g(w_2), g(w_3)) \times \Psi(f(x_{11}, x_{12}), f(x_{21}, x_{22}), g(y_1), g(y_2))$ for the points (w_1, w_2, w_3) satisfying $w_1 = w_2 = w_3 = S$ or $w_1 + w_2 = S, w_3 = S$.

	Case 1	Case 2	Case 3
$w_1 = w_2 = w_3 = S$	$\Phi_4(f(z_{21}, M))$	$\Phi_5(f(z_{21}, M))\Psi_1(f(z_{21}, M))$	$\Phi_5(f(z_{21}, M))\Psi_1(f(z_{21}, M))$,
$w_1 + w_2 = S, w_3 = S$	$\Phi_1(f(z_{21}, M))\alpha_1$	$\Phi_3(f(z_{21}, M))\Psi_2(f(z_{21}, M))$	$\Phi_2(f(z_{21}, M))\Psi_1(f(z_{21}, M))$

Case 1 means $x_1 = z_1 + z_2, x_2 = z_3, y_1 = w_1 + w_2, y_2 = w_3$. Case 2 means $x_1 = z_1 + z_3, x_2 = z_2, y_1 = w_1 + w_3, y_2 = w_2$. Case 3 means $x_1 = z_2 + z_3, x_2 = z_1, y_1 = w_2 + w_3, y_2 = w_1$.

$$\alpha_1 = \frac{1-u}{1+(N-1)u}, \Phi_1(f) = \frac{(2-u)(1-v+vf)}{2+\frac{2(N-2)u}{3}+\frac{(N-2)(2-u)v}{3}(1-f)}, \Phi_2(f) = \frac{(1-u)(2-v+vf)}{2+\frac{2(N-2)u}{3}+\frac{(N-2)(2-u)v}{3}(1-f)},$$
$$\Phi_3(f) = \frac{2-u-v+vf}{2+\frac{2(N-2)u}{3}+\frac{(N-2)(2-u)v}{3}(1-f)}, \Phi_4(f) = \frac{1-v+vf}{1+\frac{(N-2)v}{3}(1-f)}, \Phi_5(f) = \frac{2-v+vf}{2+\frac{2(N-2)v}{3}(1-f)}.$$

V. FIGURE S1 AND S2

Figure S1 shows that the division of plane (v, u) based on the optimal migration ranges of the two layers r_1^* and r_2^* when the two layers use the same game. A population of size $N = 100$ is distributed in the two network layers, each of which assumes $M = 10$, $M = 30$, or $M = 50$ communities. The proportion of the cooperative level of the first layer in the overall cooperative level $\omega = 0.5$. In (A–C), For small migration probabilities v or large mutation probabilities u, any migration ranges of the two layers can't induce natural selection to favor cooperation over defection. For other probabilities u and v, the optimal migration ranges of the two layers r_1^* and r_2^* are both identical to the value of r which can be the largest ($r = M/2$), some intermediate ($r \in \{2, 3, \cdots, M/2 - 1\}$), or the smallest range ($r = 1$). As v grows, r_1^* and r_2^* become smaller. In (D–F), the two layers use the snowdrift game. The phenomenon disappears that any migration ranges of the two layers can't lead natural selection to favor cooperation. The optimal migration ranges r_1^* and r_2^* are still identical and are located successively in the largest, some intermediate, and the smallest range as v grows, just like the case for the prisoner's dilemma. The division of the plane (v, u) is greatly affected by the community number of each layer M.

Figure S2 shows that the division of plane (v, u) based on the optimal migration ranges of the two layers r_1^* and r_2^* when the first layer uses the prisoner's dilemma and the second the snowdrift game. A population of size $N = 100$ is distributed in the two network layers, each of which assumes $M = 10$, $M = 30$, or $M = 50$ communities. We study three cases $\omega = 0.2$, $\omega = 0.5$, and $\omega = 0.8$, where ω is the proportion of the cooperative level of the first layer in the overall

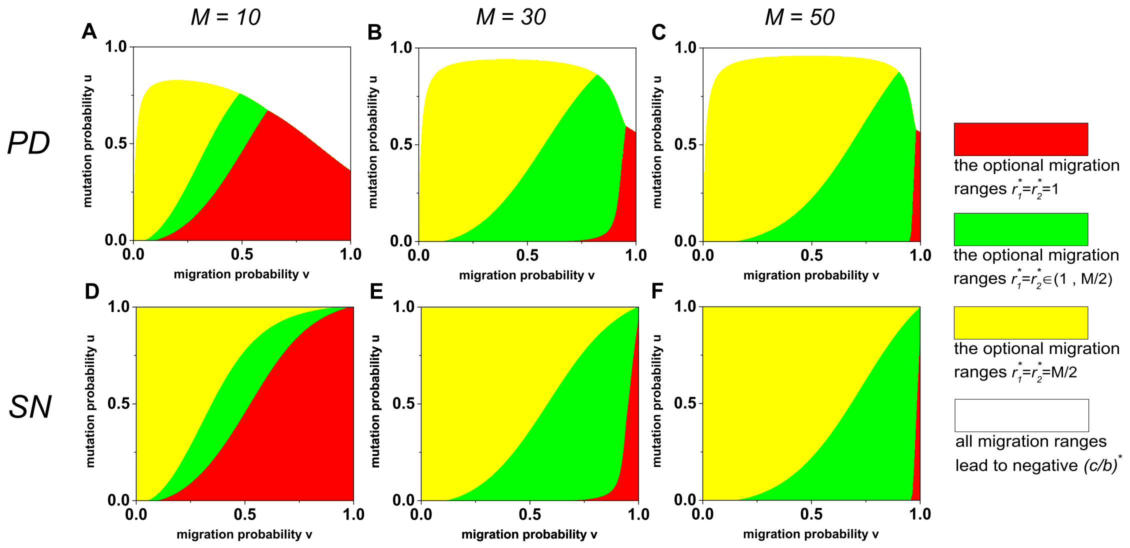

FIG. S1: When the two network layers use the same game, the division of the plane (v, u) based on the optimal migration ranges of the two layers r_1^* and r_2^*, which lead to the largest value of the critical cost-to-benefit ratio $(c/b)^*$ over the set $\{1, 2, \cdots, \lfloor M/2 \rfloor\} \times \{1, 2, \cdots, \lfloor M/2 \rfloor\}$. A population of size $N = 100$ is distributed in the two network layers, each of which assumes $M = 10$, $M = 30$, or $M = 50$ communities. The proportion of cooperative level of the first layer in the overall cooperative level $\omega = 0.5$. The two layers use the prisoner's dilemma (PD) in (A–C) and the snowdrift game (SN) in (D–F).

cooperative level. The insets show the division of (v, u) more clearly when v and u are both small. As the migration probability v grows, r_1^* is successively the largest ($r_1^* = M/2$), some intermediate ($r_1^* \in (1, M/2)$), and the shortest range ($r_1^* = 1$), and the appearance sequence of r_2^* depends on u. The area of (v, u) for $r_2^* = 1$ expands as M decreases or as ω increases. The division of the plane (v, u) is greatly affected by M.

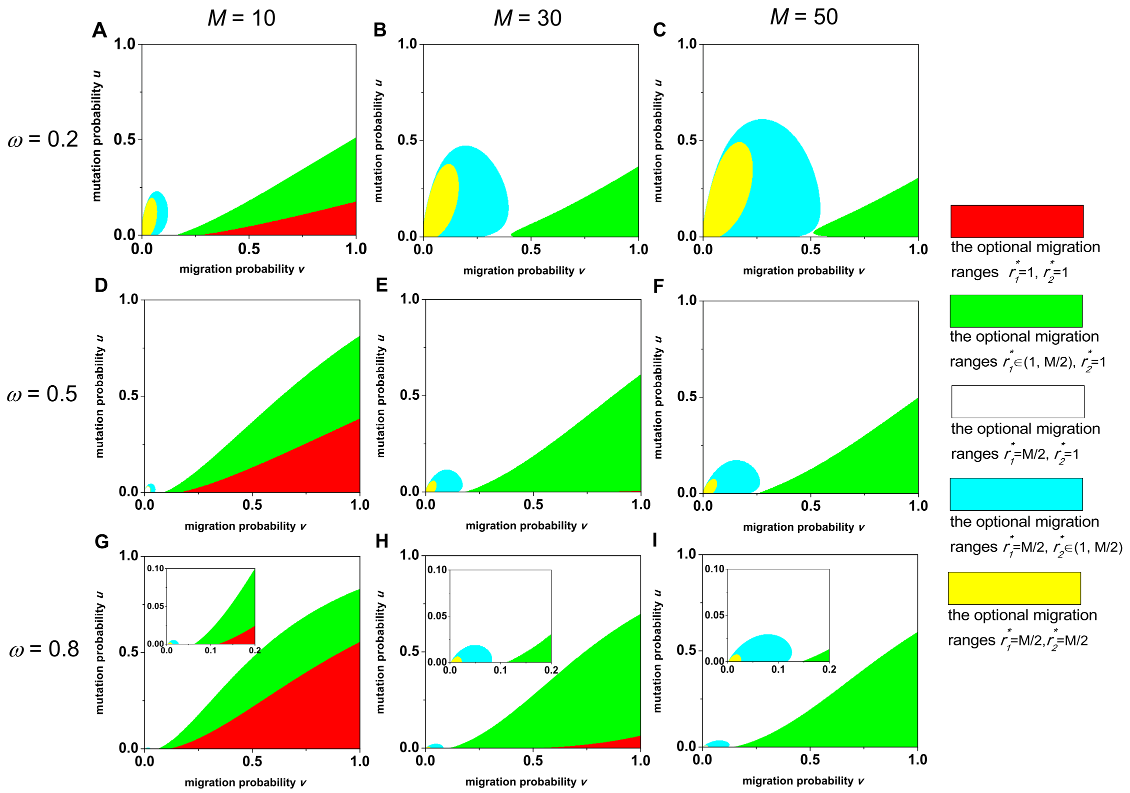

FIG. S2: When the first layer uses the prisoner's dilemma and the second the snowdrift game, the division of the plane (v, u) based on the optimal migration ranges of the two layers r_1^* and r_2^*, which lead to the largest value of the critical cost-to-benefit ratio $(c/b)^*$ over the set $\{1, 2, \cdots, \lfloor M/2 \rfloor\} \times \{1, 2, \cdots, \lfloor M/2 \rfloor\}$. A population of size $N = 100$ is distributed in the two network layers, each of which assumes $M = 10$, $M = 30$, or $M = 50$ communities. We study three cases $\omega = 0.2$, $\omega = 0.5$, and $\omega = 0.8$, where ω is the proportion of the cooperative level of the first layer in the overall cooperative level. The insets show the division of (v, u) more clearly when v and u are both small.
